# Docking protein 6 (DOK6) selectively docks the neurotrophic signaling transduction to restrain peripheral neuropathy

**DOI:** 10.1038/s41392-024-01742-2

**Published:** 2024-02-14

**Authors:** Yan Guo, Pan Xiang, Xiaojiao Sun, Wei Liu, Jiafeng Zhou, Bin Yin, Lin Hou, Boqin Qiang, Huiliang Li, Pengcheng Shu, Xiaozhong Peng

**Affiliations:** 1 Department of Molecular Biology and Biochemistry, Institute of Basic Medical Sciences, Medical Primate Research Center, Neuroscience Center, Chinese Academy of Medical Sciences, School of Basic Medicine Peking Union Medical College, Beijing, China; 2 State Key Laboratory of Common Mechanism Research for Major Diseases, Beijing, 100005 China; 3 Wolfson Institute for Biomedical Research, University College London, Gower Street, London, WC1E 6BT UK; 4 Chinese Institute for Brain Research, Beijing, 102206 China; 5 State Key Laboratory of Respiratory Health and Multimorbidity, Beijing, 100005 China; 6 Institute of Laboratory Animal Science, Chinese Academy of Medical Sciences & Peking Union Medical College, Beijing, 100021 China

**Keywords:** Peripheral nervous system, Diseases of the nervous system

## Abstract

The appropriate and specific response of nerve cells to various external cues is essential for the establishment and maintenance of neural circuits, and this process requires the proper recruitment of adaptor molecules to selectively activate downstream pathways. Here, we identified that DOK6, a member of the Dok (downstream of tyrosine kinases) family, is required for the maintenance of peripheral axons, and that loss of *Dok6* can cause typical peripheral neuropathy symptoms in mice, manifested as impaired sensory, abnormal posture, paw deformities, blocked nerve conduction, and dysmyelination. Furthermore, Dok6 is highly expressed in peripheral neurons but not in Schwann cells, and genetic deletion of *Dok6* in peripheral neurons led to typical peripheral myelin outfolding, axon destruction, and hindered retrograde axonal transport. Specifically, DOK6 acts as an adaptor protein for selectivity-mediated neurotrophic signal transduction and retrograde transport for TrkC and Ret but not for TrkA and TrkB. DOK6 interacts with certain proteins in the trafficking machinery and controls their phosphorylation, including MAP1B, Tau and Dynein for axonal transport, and specifically activates the downstream ERK1/2 kinase pathway to maintain axonal survival and homeostasis. This finding provides new clues to potential insights into the pathogenesis and treatment of hereditary peripheral neuropathies and other degenerative diseases.

## Introduction

Nearly all aspects of neural activity, from the generation of neurons and glial cells to the establishment and maintenance of proper circuitry, depend on precise intracellular signaling transduction in response to environmental cues (e.g., neurotransmitters, growth factors, hormones, and other chemical stimuli). A variety of signaling pathways are involved in the regulation of these processes, forming a sophisticated yet tightly organized network to achieve diverse functional outcomes. Receptor tyrosine kinase (RTK) signaling, in which the receptors contain a catalytic tyrosine kinase in the intracellular domain, is widely recognized to play a critical role in the development and maintenance of the peripheral nervous system (PNS), affecting both the axons of peripheral neurons and the myelin sheaths produced by Schwann cells (SCs).^1,2^ Dysregulated transduction of RTKs signaling contributes to various neuropathologies.^3,4^

RTKs constitute a large family of cell surface receptors that are typically activated by growth factors and trophic factors.^1,2^ Upon activation, RTKs recruit adapters and effector proteins to facilitate various downstream intracellular signaling cascades, including the mitogen-activated protein kinase (MAPK), phosphatidylinositol 3-kinase (PI3K), Janus kinase and signal transducer and activator of transcription (JAK/STAT), and PLCγ pathways. The MAPK cascades are subdivided into at least four subfamilies: extracellular signal-related kinases (Erk1/2), Erk5, JNK1–3, and the p38 kinases, which comprise p38α, β, γ, and δ.^1,4^ It is intriguing that multiple RTKs utilize comparable molecular subsets or overlapping downstream cascade pathways to achieve diverse biological outcomes, such as cell growth, differentiation, survival, and function. However, how signaling specificity is achieved, or how to generate a distinct cellular response, remains a significant challenge. In addition, some RTK signaling complexes are also transported, particularly the neurotrophin signaling. The specific target-derived neurotrophic factors (NTs) bind to their receptors to form signaling endosomes through receptor-mediated endocytosis, then the ligand–receptor complexes undergo retrograde transport through the microtubule-based motor system relying on dynein and Rab protein,^5–8^ and recruit downstream molecules to activate specific downstream pathways.^7,8^ It is noteworthy that mutations in NGF and TrkA, or defects in components of the retrograde axonal transport pathway (e.g., *DYNC1H1, RAB7A, KIF1A* and *DCTN1*), can result in hereditary peripheral neuropathies, which have a broad spectrum with variable sensory, motor, autonomic, and other organ trophic symptoms depending on the type of neurons involved and lead to damage of these fibers.^9–14^ The trafficking of signaling molecules controls their precise spatial location for transduction events, and signaling cascades also influence trafficking. However, the intricate relationship between the regulation of these two signaling events remains incompletely understood. Understanding the components of signal transduction is crucial to unraveling the underlying mechanisms, as their concentration, localization, and interactions with other proteins are critical for determining distinct and context-specific responses.

Adapter proteins, also known as docking proteins,^15^ play a critical role in RTK-mediated signaling pathways by precisely recruiting specific molecules and governing signaling cascades.^15–17^ Acting as a scaffold, they interact with various protein binding partners, facilitating the formation of extensive signaling complexes. Additionally, adapter proteins modulate the dynamic activation of the signaling pathway.^18^ Different cells may concurrently and/or differently employ multiple adapter proteins to selectively regulate distinct downstream targets. In essence, these adapter proteins serve as significant nodes for neuronal cells to effectively respond to external signals. Nevertheless, the functionality of many adapter proteins remains to be elucidated. DOK6, a member of the downstream tyrosine kinase (DOK) family protein, is expressed predominantly in the nervous system.^19^ It comprises the typical structural features characterized by N-terminal pleckstrin-homology (PH) and phosphotyrosine-binding (PTB) domains, as well as multiple tyrosine phosphorylation sites in C-terminal.^20^ Although members of the DOK family share similar structural domains, their functions are distinct, and some of them even have opposing functions. However, to date, the in vivo role of *Dok6* remains unknown. Crowder *et al*. showed that DOK6 acts as a substrate of RET signaling and facilitates Ret-mediated neurite outgrowth in N2A-α1 cells.^19^ Our previous work has shown that DOK6 selectively binds to TrkC receptors through its PTB domain, dependent on kinase activity, and participates in NT-3-mediated neurite outgrowth in the mouse cortical neurons.^21^ A recent study indicates that *Dok6* polymorphisms significantly increase susceptibility to Hirschsprung’s disease, a developmental defect that is defined by the absence of enteric ganglia in the distal colon, suggesting a role for Dok6 in PNS development.^22^

In this study, we identified DOK6 as a novel factor involved in the maintenance of peripheral axons, and found that loss of *Dok6* leads to typical symptoms of peripheral neuropathy in mice. Notably, Dok6 is specifically expressed in certain types of DRG neurons, but not in SCs in the PNS, is required for the myelination of axons. Mechanistically, DOK6 acts as a key adapter protein to facilitate the retrograde transport of NT-Trk signaling and to selectively activate the MEK/ERK signaling pathway. This finding also provides new insight into the underlying mechanisms that drive peripheral neuropathy.

## Results

### The absence of *Dok6* in mice leads to motor abnormalities and nociceptive hyposensitivity

To investigate the function of *Dok6*, we first crossed floxed *Dok6* (*Dok6*^*fl/fl*^) mice with *EIIA-cre* mice to generate *Dok6*^*−/−*^ mice (Supplementary Fig. S1a–c) since *EIIA-cre* mice activate Cre activity in early fertilized eggs.^23^ After confirming that DOK6 is predominantly expressed in the brain (Supplementary Fig. S1d), we further verified the conventional deletion of the Dok6 gene at the protein level (Supplementary Fig. S1e).

**Fig. 1:**
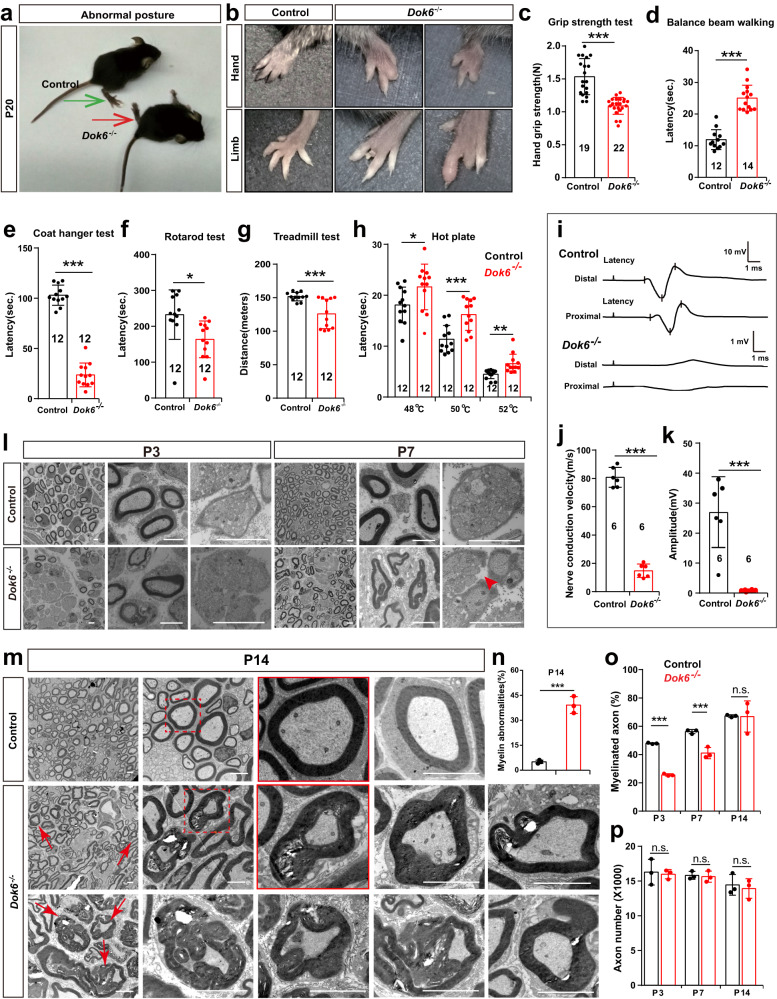
Motor abnormality, nociceptive hyposensitivity, and peripheral myelin abnormalities in *Dok6*^*−/−*^ mice**. a**, **b** *Dok6*^*−/−*^ mice exhibited abnormal posture and aberrant gait (arrows). Severe injury and deformity of paws in both forelimbs (upper) and hindlimbs (lower) of *Dok6*^−/−^ mice were observed at P20. **c–h** Motor performance was examined with the grip strength test, beam balance test, coat hanger test, rotarod test, and treadmill test in age-matched wild-type control group mice and *Dok6*^−/−^ mice at P60. Nociceptive sensitivity was measured by the hot plate test. **i** conduction velocity recording of CAPs with stimulated right sciatic nerves from the control group and *Dok6*^−/−^ mice at age P60. Representative traces from measurements per genotype are shown. **j**, **k** Nerve conduction velocity and amplitude in control and *Dok6*^−/−^ mice. **l** The ultrastructure of the sciatic nerve was observed with electron microscopy in the control group and *Dok6*^*−/−*^ mice at P3 and P7 (*n* = 3 per genotype). At P3 in the *Dok6*^−/−^ sciatic nerve, delayed axonal sorting by SC. At P7, several axons were ensheathed by promyelinating SCs, abnormal folding was observed in the *Dok6*^−/−^ sciatic nerve, and an abnormal Remak Schwann cell was present (arrowhead). Scale bars, 2 μm (Lanes 3 and 6, Scale bars, 500 nm). **m** The ultrastructure of the sciatic nerve was observed with electron microscopy in the control group and *Dok6*^−/−^ mice at P14. Misfolded myelin, focal hypermyelination, myelin debris, such as myelin tomacula and invaginating recurrent loops were detected in *Dok6*^−/−^ mice axons (arrow). Scale bars, 2 μm. **n** Quantitation of sciatic nerve myelin abnormalities in the control and *Dok6*^−/−^ mice at P14 (*n* = 3 animals per genotype, on average 7 randomly taken electron micrographs at the same magnification per animal were chosen. The percentage of abnormal myelin significantly increased in *Dok6*^−/−^ mice. **o** The percentage of myelinated axons in the P3 (*n* = 3 per genotype, on average nine sections per animal), P7 (*n* = 3 per genotype, on average 7 sections per animal), and P14 (*n* = 3 per genotype, on average 7 sections per animal) control, *Dok6*^*−/−*^ mice sciatic nerve. Randomly taken electron micrographs at the same magnification per animal were chosen. **p** Total axon number in the P3, P7, and P14 control, *Dok6*^*−/−*^ mice sciatic nerve. Data are presented as the mean ± SD, *p < 0.05, **p < 0.01, ***p < 0.001, two-tailed unpaired Student’s *t* test

Intercrosses between *Dok6*^*+/-*^ heterozygotes yielded ~25% *Dok6*^*−/−*^ pups after birth, indicating that *Dok6*^*−/−*^ did not affect survival at birth, nor did it affect later survival (Supplementary Fig. S1f). However, *Dok6*^*−/−*^ mice were distinguishable from the control group and heterozygous littermates based on abnormal posture of the forelimbs and hindlimbs. *Dok6*^*−/−*^ pups appeared to walk abnormally with staggered gait and abnormal posture at postnatal day 20 (P20) (Fig. 1a, Supplementary Video S1). A total of 2.6% *Dok6*^*+/-*^ mice had an abnormal gait phenotype (Supplementary Table S1), while 31.1% *Dok6*^*−/−*^ mice showed abnormal hindlimb deficiency and swelling (Fig. 1b, Supplementary Table S1), whereas the littermate control did not display similar phenotypes.

We then assessed the motor coordination ability, pain, and temperature sensing abilities of age-matched wild-type control group mice and *Dok6*^*−/−*^ mice. Grip strength was reduced in *Dok6*^*−/−*^ mice compared to the control group (Fig. 1c). Control group mice required less time to cross the horizontal bar than did *Dok6*^*−/−*^ mice (Fig. 1d). Additionally, *Dok6*^*−/−*^ mice exhibited a reduced duration on the coat hanger after being placed in the middle of the lower horizontal bar (Fig. 1e). The rotarod test was used to evaluate motor coordination capability and there was a significant decrease in rotarod residence time in *Dok6*^*−/−*^ mice compared to control mice (Fig. 1f). Moreover, treadmill distance and stimulation times were recorded for all mice, and the *Dok6*^*−/−*^ group had reduced treadmill lane distance (Fig. 1g). These tests all indicated that *Dok6*^*−/−*^ mice had impaired motor function. Meanwhile, the results showed that *Dok6*^*−/−*^ mice had a longer incubation period than the control group at 48 °C, 50 °C and 52 °C (Fig. 1h), and *Dok6*^*−/−*^ mice were less susceptible to noxious stimulation than the control group.

The altered sensory symptoms and motor dysfunction observed in *Dok6*^*−/−*^ mice suggest abnormalities in axonal function. Since slowed motor nerve conduction velocity (NCV) in the sciatic nerve is one of the key electrophysiological features of axonal function, we recorded NCV, the speed at which an action potential is transmitted along the axon, and the amplitude of the compound action potentials (CAPs) in the sciatic nerve of *Dok6*^*−/−*^ mice and control mice. Stimulation at proximal sites in the sciatic nerve revealed flattened potential peaks, reduced NCV and amplitude in the sciatic nerve of *Dok6*^*−/−*^ mice (Fig. 1i-k), indicating severe axonal damage caused by *Dok6* knockout.

### Neuronal fate specification was unchanged but impaired the survival of DRG sensory neurons in *Dok6* deletion mice

*Dok6*^*−/−*^ mice showed abnormal pain and temperature sensation, hindlimb deficiency, and swelling abnormalities, indicating defects in peripheral nerve circuitry. Meanwhile, our previous work has shown that DOK6 is able to interact with the TrkC receptor to mediate neurotrophin signaling.^21^ Therefore, we wondered whether the functional defects caused by *Dok6* deletion were due to the abnormal fate specialization of sensory neurons or neuronal apoptosis caused by blockade of neurotrophic factor signals. To test this possibility, we performed in situ hybridizations on transverse lumbar L4-L5 sections of wild-type and *Dok6*^*−/−*^ mice at early stage E16.5 and at the mature stage P21 (Supplementary Fig. S2a-b). The number of nociceptive neurons (*TrkA*^+^, *CGRP*^+^), mechanoreceptor neurons (*TrkB*^+^ or *Ret*^+^) that convey touch sensation, and proprioceptive neurons (*TrkC*^*+*^, *Runx3*^*+*^, *PV*^*+*^) that sense limb movement and position.^24^ were counted. Embryonic *TrkA*^*+*^ neurons generate adult *TrkA*^*+*^ peptidergic and *Ret*^+^ nonpeptidergic neurons.^25^ The number of most DRG neurons appeared unchanged in *Dok6*^*−/−*^ mice compared to control mice at E16.5. Nonetheless, the number of proprioceptive neurons (*TrkC*^*+*^, *Runx3*^*+*^, *PV*^*+*^) and *Ret*^*+*^ neurons exhibited a slight reduction in the *Dok6*^*−/−*^ DRG at P21 (Supplementary Fig. S2c, d). This suggests that *Dok6* deletion does not alter early neuronal fate specification, but impaired the survival of some subtypes of DRG sensory neurons.

### *Dok6*^*−/−*^ mice exhibit peripheral myelin abnormalities

Since the structural integrity of the myelin sheath is required for functional sensation in the PNS, we next examined whether the myelin structure was altered in *Dok6*^*−/−*^ mice. The myelinated fibers in the sciatic nerve of the littermate control group mice and the *Dok6*^*−/−*^ group were examined by transmission electron microscopy (TEM). In the sciatic nerve of P21 *Dok6*^*−/−*^ mice, there was a large amount of dysmyelination, including comma-shaped outfolding or recurrent loop abnormalities or tomaculum with myelin breakdown (data not shown). Therefore, we analyzed the different stages at the earlier stage. The process by which Schwann cells select larger axons for myelination during development is known as radial sorting.^26^ At P3, radial sorting of axons by SCs was delayed in the *Dok6*^*−/−*^ sciatic nerve (Fig. 1l, m). A few axons were ensheathed by promyelinating SCs, and abnormal multilayered myelin sheath structures were formed but not tightly attached to the axons in the *Dok6*^−/−^ sciatic nerve at P7. In addition, abnormal folding was detected in the *Dok6*^*−/−*^ sciatic nerve at P7 (Fig. 1l). At P14, misfolded myelin, focal hypermyelination, myelin debris, aberrant outfoldings and invaginating recurrent loops in axons were widely observed in the *Dok6*^*−/−*^ sciatic nerve (Fig. 1m). The ratio of myelinated axons was almost unchanged in *Dok6*^*−/−*^ mice compared to control group mice at P14 (Fig. 1n–p), and the percentage of sciatic nerve axonal myelin abnormalities was significantly increased in *Dok6*^−/−^ mice (Fig. 1n). In summary, *Dok6* deletion in mice results in a typical peripheral neuropathy, including decreased nociceptive and proprioceptive sensitivity, reduced nerve conduction velocities and abnormal peripheral myelination.

### *Dok6* is expressed specifically in dorsal root ganglion (DRG) neurons in the PNS

**Fig. 2:**
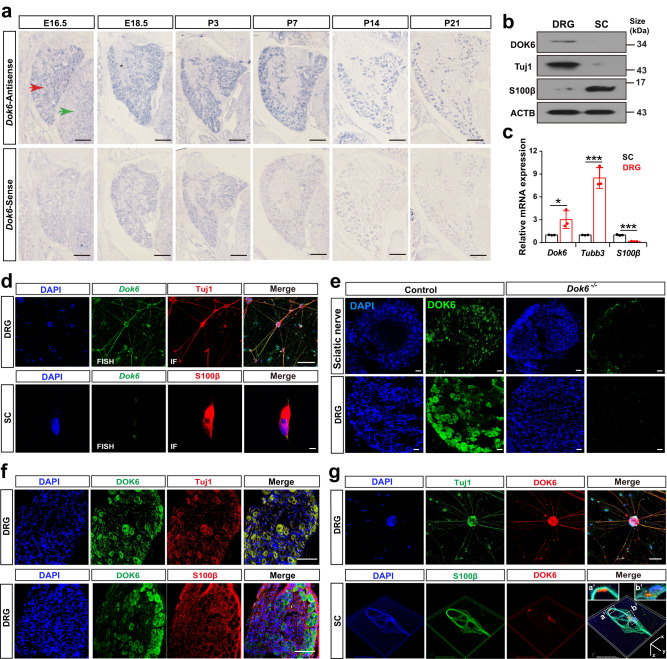
Dok6 was expressed in DRG neurons. **a** *Dok6* RNA in situ hybridization with tissue from DRG tissues collected at E16.5, E18.5, P3, P7, and P14. *Dok6* expression started in the DRG at E16.5, reached a peak at E18.5, and then gradually decreased after birth. The red arrow points to the DRG and the green arrow points to the spinal cord. A *Dok6*-sense riboprobe was used as the negative control. **b** Western blot assays for DOK6 with protein lysates from cultured DRG neurons and SCs. DOK6 was predominantly detected in DRG neurons. **c** qRT–PCR assays with isolated DRG neurons and SCs. *Dok6* was mainly detected in DRG neurons. qRT–PCR for *Tubb3* and *S100β* showed a high purity of isolated cells. **d** Histochemical assays with the DRG neuron-SC coculture system. Cultured cells were subjected to fluorescent in situ hybridization for *Dok6* (green), followed by immunostaining for Tuj1 (red) or S100β (red). **e** Immunostaining of customized antibody to Dok6 in P7 age-matched wild-type control group and mutant mice sciatic nerve and DRG tissue to confirm its expression, Scale bars, 100 μm. **f** Histochemical assays of the DRG tissue, co-labeling of DOK6(green) with neuronal cell marker Tuj1 (red) and Schwann cell marker S100β (red), Scale bar, 100 μm. **g** Histochemical assays of the DRG neuron and SC with Dok6. Cultured cells were subjected to immunofluorescence for DOK6 (red), followed by immunostaining for Tuj1 (green) or S100β (green). Scale bar: up panel = 100 μm, low panel: *x* = 1 μm, *y* = 1 μm, *z* = 5 μm. Data are presented as the mean ± SD, *p < 0.05, **p < 0.01, ***p < 0.001, two-tailed unpaired Student’s *t* test

Peripheral neuropathy can be caused by myelinopathy or neuronopathy.^27^ To further distinguish whether DOK6 acts on neurons or SCs to cause peripheral neuropathy, we utilized in situ hybridization to analyze the temporal and spatial expression patterns of *Dok6* mRNA. The results showed that *Dok6* was specifically expressed in the nervous system from the early stages of neurodevelopment, especially in the dorsal root ganglion (DRG) (Supplementary Fig. S1g). Next, we analyzed the expression dynamics of Dok6 in the dorsal root ganglia at E16.5, E18.5, P3, P7, and P14, and found that *Dok6* continued to be highly expressed in the DRG, and its distribution ratio gradually decreased after birth (Fig. 2a).

To further investigate the cellular localization of DOK6, we detected its expression in DRG neurons and SCs cells by isolating and purifying of these two cell types from adult Sprague-Dawley (SD) rats, respectively. After confirming the purity of the cells using markers of neurons or SCs (data not shown), we performed Western blot and quantitative real-time PCR (RT-qPCR) assays to reveal that *Dok6* is predominantly expressed in DRG neurons (Fig. 2b, c). Furthermore, we used fluorescence in situ hybridization followed by immunostaining to confirm that *Dok6* was highly expressed in Tuj1^+^ DRG neurons but not S100β^+^ SCs in cultured DRGs and SCs (Fig. 2d). To validate the expression characteristic of DOK6, we generated a polyclonal antibody that specifically recognizes mouse DOK6 protein and can be used for immunostaining. While DOK6 protein was detected in the DRG and sciatic nerve of wild-type control group mice at P7, no expression of the protein was detected in the DRG or sciatic nerve of the *Dok6* KO mice (Fig. 2e). Next, we performed immunofluorescence colocalization analysis in DRG tissues (Fig. 2f) and purified culture cells (Fig. 2g), and found that DOK6 protein expression overlapped with Tuj1 expression, but not with S100β (Fig. 2f, g). It is further confirmed that DOK6 was enriched in neurons rather than in SC cells.

### *Dok6* conditional knockout (cKO) in DRG neurons but not SCs leads to peripheral myelin abnormalities

**Fig. 3:**
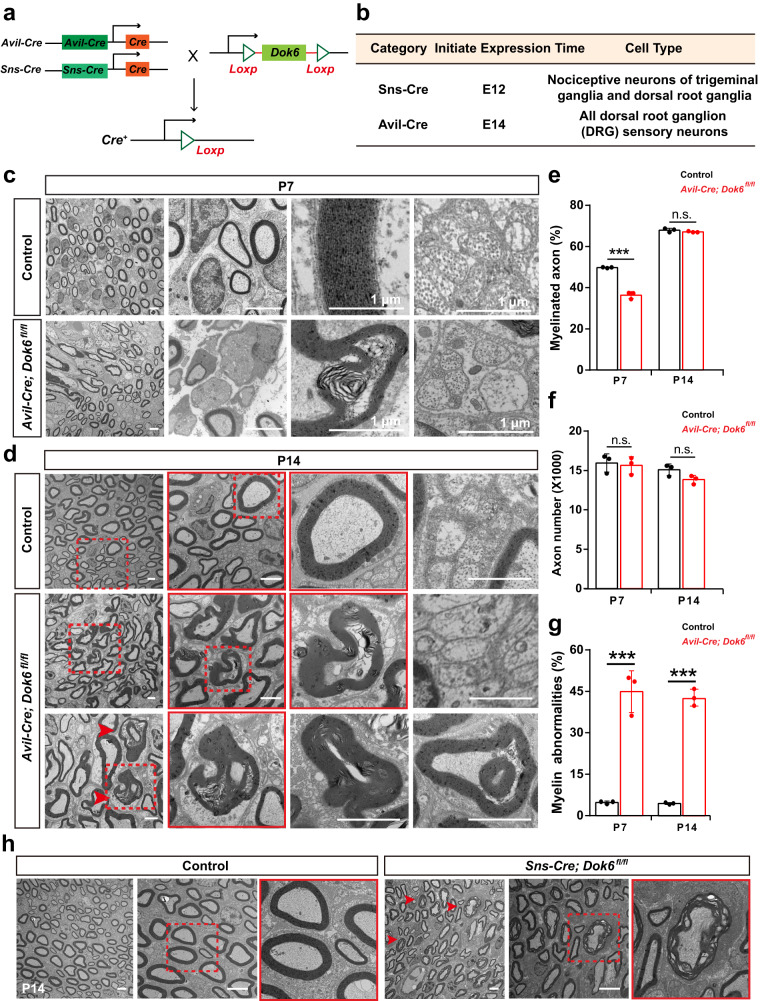
Mice lacking *Dok6* in DRG neurons showed peripheral myelin abnormalities. **a** Schematic representation of the constructs used to create the transgenic mice. **b** Two kinds of Cre tool mouse information, *Sns*, and *Avil*, are specifically expressed in neurons. *Sns* is mainly expressed in most nociceptors, and *Avil* has a range of expression and targets all subsets of peripheral sensory neurons. **c** The ultrastructure of the sciatic nerve observed with TEM in age-matched wild-type control group mice and *Avil-Cre*; *Dok6* ^*fl/fl*^ mice at P7. Degenerating myelin (arrowhead) was present in *Avil-*Cre; *Dok6* ^*fl/fl*^ mice. Scale bar, 2 μm (panels 3 and 4, Scale bar, 1 μm). **d** The ultrastructure of the sciatic nerve observed with TEM in control and *Avil-Cre*; *Dok6* ^*fl/fl*^ mice at P14. Myelin sheaths with dense degeneration and other abnormal phenotypes were present in *Avil-Cre*; *Dok6* ^*fl/fl*^ mice. Scale bar, 2 μm. **e** The percentage of myelinated axons in the P7 (*n* = 3 animals per genotype, on average 17 electron micrographs per animal) and P14 (*n* = 3 animals per genotype, on average 25 electron micrographs per animal) control, *Avil-Cre*; *Dok6* ^*fl/fl*^ mice sciatic nerve (*n* = 3 per genotype). Randomly taken electron micrographs at the same magnification per animal were chosen. **f** Total axon number in the P7 and P14 control, *Avil-Cre*; *Dok6* ^*fl/fl*^ mice sciatic nerve. **g** Quantitation of sciatic nerve myelin abnormalities in control and *Avil-Cre*; *Dok6* ^*fl/fl*^ mice at P7 (*n* = 3 animals per genotype, on average 17 electron micrographs per animal) and P14 (*n* = 3 animals per genotype, on average 25 electron micrographs per animal). Randomly taken electron micrographs at the same magnification per animal were chosen. **h** The ultrastructure of the sciatic nerve observed with TEM in control and *Sns-cre; Dok6* ^*fl/fl*^ mice at P14. Abnormal myelin sheaths were detected in *Sns-cre; Dok6* ^*fl/fl*^ mice. Scale bar, 2 μm. Data are presented as the mean ± SD, ***p < 0.001, two-tailed unpaired Student’s t test

**Fig. 4:**
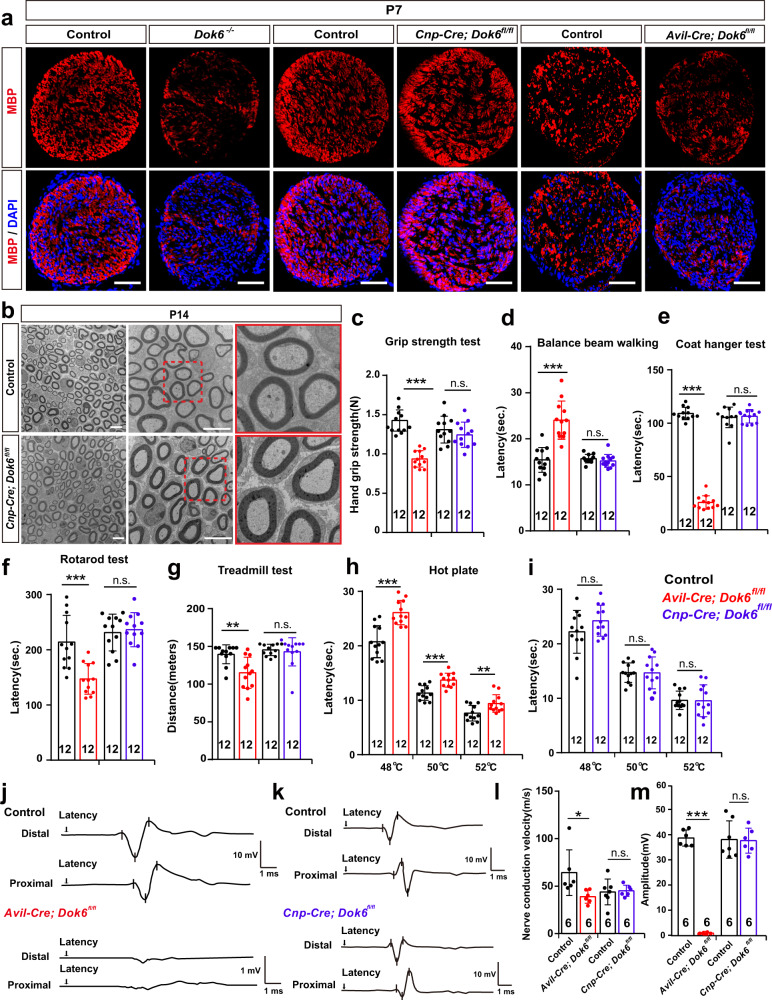
Motor abnormality, nociceptive hypersensitivity, and peripheral myelin abnormalities in *Avil-Cre; Dok6*^*fl/fl*^ rather than in *Cnp-Cre; Dok6*^*fl/fl*^ mice. **a** MBP immunostaining (red) in the P7 littermate control, *Dok6* mutant, *Cnp-cre; Dok6* ^*fl/fl*^ and *Avil-Cre; Dok6*^*fl/fl*^ mice sciatic nerve. Scale bar, 10 μm. **b** The ultrastructure of the sciatic nerve observed with TEM in Control and *Cnp-cre; Dok6* ^*fl/fl*^ mice at P14. Scale bar, 10 μm. **c–i** Motor performance was examined with the grip strength test, beam balance test, coat hanger test, rotarod test, and treadmill test in control, *Avil-Cre; Dok6*^*fl/fl*^ and *Cnp-Cre; Dok6*^*fl/fl*^ mice at P60. Nociceptive sensitivity was measured by the hot plate test. **j**, **k** Conduction velocity recording of CAPs with stimulated right sciatic nerves from Control, *Avil-Cre; Dok6*^*fl/fl*^ and *Cnp-Cre; Dok6*^*fl/fl*^ mice at P60. Representative traces from measurements per genotype are shown. **l**, **m** Nerve conduction velocity and amplitude in control, *Avil-Cre; Dok6*^*fl/fl*^ and *Cnp-Cre; Dok6*^*fl/fl*^ mice. Data are presented as the mean ± SD, *p < 0.05, **p < 0.01, ***p < *0.001*, two-tailed unpaired Student’s *t* test

To further verify whether DOK6 acts on neurons or SCs, we conditionally knocked out *Dok6* in SCs with *Cnp-Cre*.^28^ or DRG neurons with *Sns-Cre*.^29^ and *Advillin-Cre (Avil-Cre)*.^30^ (Fig. 3a, b, Supplementary Fig. S3a–g). In the *Cnp-Cre*; *Dok6*^*fl/fl*^ mice, immunofluorescence staining for MBP, a marker of mature SC, did not reveal significant changes in myelin density or the number of myelinated axons in the sciatic nerve (Fig. 4a, Supplementary Fig. S3h). TEM observation revealed that there was no obvious microstructural difference between sciatic nerves in *Cnp-Cre*; *Dok6*^*fl/fl*^ mice and littermate control group mice (Fig. 4b). However, in *Avil-Cre*; *Dok6*^*fl/fl*^ mice, in which cre targets all subsets of peripheral sensory neurons, the sciatic nerve did show significant signs of myelin sheath degradation or misfolded myelin as early as P7 (Fig. 3c). Myelin sheaths in *Avil-Cre; Dok6*^*fl/fl*^ mice at P14 were generally not compact and displayed myelin fragments, onion-like, and comma-shaped outfolding configurations, splitting of the myelin lamellae with dense degeneration (Fig. 3d). The ratio of myelinated axons reduced in *Avil-cre; Dok6*^*fl/fl*^ mice at P7 rather than at P14 (Fig. 3e, f), and the percentage of sciatic nerve axonal myelin abnormalities was significantly increased in *Avil-Cre; Dok6* ^*fl/fl*^ mice at P7 and P14 (Fig. 3g). Myelin labeled by MBP staining was found to be significantly decreased in the *Avil-Cre*; *Dok6*^*fl/fl*^ sciatic nerve (Fig. 4a, Supplementary Fig. S3h). Furthermore, the thickness of axon myelin sheaths of different diameters was measured in *Avil-Cre; Dok6*^*fl/fl*^ mice and littermate control group mice. The average g-ratio in the sciatic axons of *Avil-Cre; Dok6*^*fl/fl*^ mice and the relationship between the g-ratio and sciatic nerve axon diameter were significantly decreased, which are similar to the *Dok6*^*−/−*^ mice group at P14 (Supplementary Fig. S3i-p). This suggests the average myelin thickness in *the Avil-Cre; Dok6*^*fl/fl*^ sciatic nerve was increased. Additionally, similar peripheral myelin abnormalities were also observed in *Sns-cre*; *Dok6* ^*fl/fl*^ mice at P14 (Fig. 3h).

Besides, to further verify how DOK6 affects the peripheral nervous system, we performed behavioral and electrophysiological analysis in these different lines of *Dok6* knockout mice at the same age as in *Dok6* full KO. The data show that *Dok6* mutation in DRG neurons with *Avil-Cre* but not *Dok6* knockout in SCs with *Cnp-Cre*, leads to typical conduction velocity changes in mice, similar to those of *Dok6* conditional knockout (Fig. 4c–m), strongly suggesting that *Dok6* expression in DRG neurons is essential for PNS myelination in mice. In short, the abnormal myelination phenotype in *Dok6*^*−/−*^ mice may not be directly caused by *Dok6* function in SCs and could be the indirect consequence of axonal pathological changes.

### Mice lacking *Dok6* in DRG neurons exhibit axonal degeneration and axonal retrograde transport defects

**Fig. 5:**
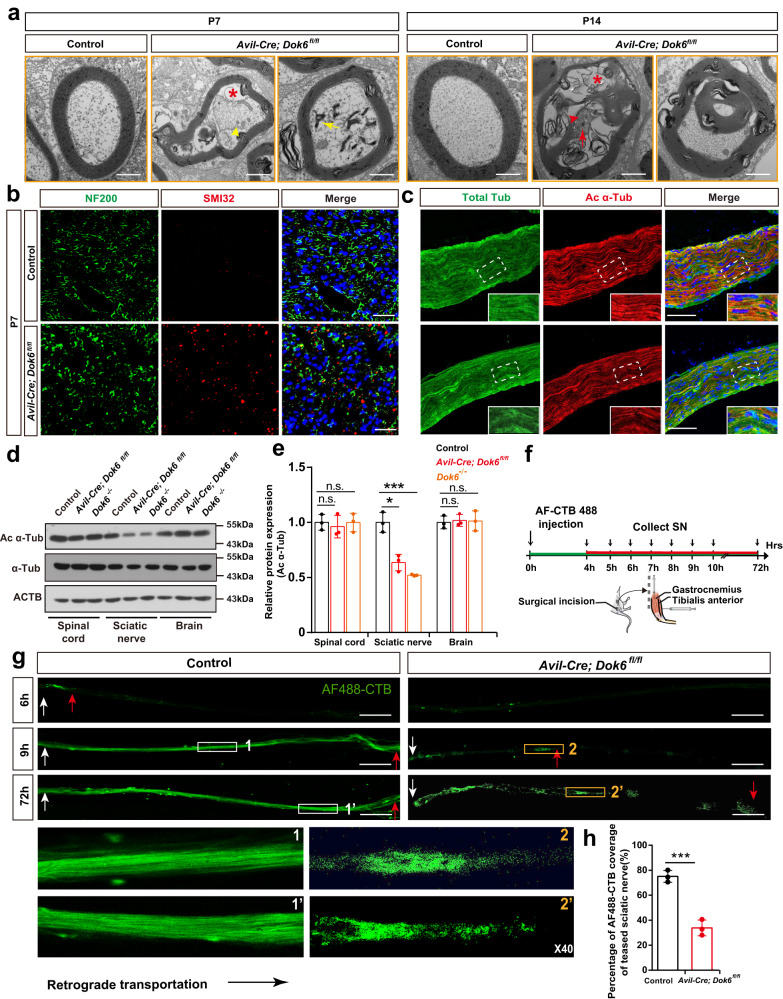
Mice lacking *Dok6* in DRG neurons exhibited axonal degeneration and axonal retrograde transport abnormalities. **a** Ultrastructure of the sciatic nerve observed with TEM in littermate control and *Avil-Cre*; *Dok6* ^*fl/fl*^ mice at P7 and P14. Axon pathological alterations were present in *Avil-Cre*; *Dok6* ^*fl/fl*^ mice. Vacuoles are on the perimeter of the axon (red asterisk), fiber with dark cytoplasm (yellow arrowhead) at the periphery of the axon, the axoplasmic contents appear to be mitochondria or other membrane-bound organelles (yellow arrow); the axoplasm contains an accumulation of mitochondria as well as a few lysosomes (red arrow and arrowhead), detachment of the myelin sheath from the axon and degenerated axon presented with vacuoles (red asterisk) and axon loss. Scale bar, 500 nm. **b** Immunostaining of NF200 (green, a marker for axons) and SMI32 (red, a marker for damaged axons) in the sciatic nerve of control and *Avil-Cre*; *Dok6*^*fl/fl*^ mice at P7. Scale bar, 100 μm. **c** Immunostaining of tubulin (green, a marker for axons) and acetylated α-tubulin (red, a marker for damaged axon tubulins) in the sciatic nerve of control and *Avil-Cre*; *Dok6*^*fl/fl*^ mice at P7. Scale bar, 100 μm. **d**, **e** Western blot analysis showing decreased α-tubulin acetylation in sciatic nerves of *Avil-Cre*; *Dok6*^*fl/fl*^ mice. Data are presented as the mean ± SD, *p < 0.05, ***p < 0.001, One-way ANOVA between different lines of mice. **f** Schematic diagram for AF488-CTB injection into the muscle. Inject the lateral head of the gastrocnemius using a steep angle from the plane of the table and use a much shallower, almost horizontal, angle (10–20°) for the tibialis anterior. **g** AF488-CTB was used as a tracer to investigate sciatic nerve retrograde transport in control and *Avil-Cre*; *Dok*6^*fl/fl*^ mice. The left end of each diagram represents the distal end of the teased sciatic nerve. Scale bar, 500 μm. **h** Histograms showing axonal transport parameters of AF488-CTB to analyze the percentages of AF488-CTB coverage in teased sciatic nerve by the end of 9 h from injection time point in control and *Avil-Cre*; *Dok*6^*fl/fl*^ mice. Data are presented as the mean ± SD, ***p < 0.001, two-tailed unpaired Student’s *t* test

Electron microscopy data revealed several feature changes of axonal degeneration in the *Dok6*^*−/−*^ sciatic nerve, such as axon shrinkage or axonal loss (empty myelin sheath), accumulation of mitochondria and lysosomes in the axoplasm, and splitting or contortion of the remaining myelin sheath (Supplementary Fig. S4a), strongly suggesting nerve degeneration. Moreover, we also found typical electron microscopic features of peripheral nerve axonal degeneration in *Avil-Cre; Dok6*^*fl/fl*^ sciatic nerve axons at P7 and P14, such as splitting of the myelin sheath, dense cytoplasm with vacuole, distorted lamellae separated from the axonal membrane, which are similar to the *Dok6*^*−/−*^ (Fig. 5a).

To further confirm the occurrence of axonal degeneration upon loss of *Dok6*, we detected SMI32^+^, a marker of damaged axons that labels non-phosphorylated neurofilaments,^31^ by immunostaining in *Dok6* deletion sciatic nerves. The number of SMI32^+^ axons was significantly increased in the sciatic nerve of *Avil-Cre*; *Dok6*^*fl/fl*^ mice at P7 compared to the littermate wild-type control group (Fig. 5b, Supplementary Fig. S4b). Furthermore, we detected the expression of acetylated α-tubulin (ac α-tub), a marker of stable microtubules,^14^ in different tissues in *Dok6* deletion PNS. Immunostaining showed a significant decrease in acetylated α-tubulin expression in the sciatic nerves of *Avil-Cre*; *Dok6*^*fl/fl*^ mice, and the decrease in acetylated α-tubulin indicated a defect in the axons (Fig. 5c, Supplementary Fig. S4c). Western blot analysis showed that the decrease in acetylated α-tubulin was specific to sciatic nerves in both *Avil-Cre*; *Dok6*^*fl/fl*^ mice and *Dok6*^*−/−*^ mice, whereas no obvious change was found in spinal cord and brain tissues for both α-tubulin and acetylated α-tubulin levels (Fig. 5d, e). Next, to determine whether *Dok6* deletion-induced axonal degeneration affects axonal retrograde transport, the retrograde tracer AF488-CTB was injected into the mouse gastrocnemius and the tibialis anterior, and a fixed position of the sciatic nerve was separated and analyzed after a few hours (Fig. 5f–h). In the control group, we found that AF488-CTB was transported to the distal end of the fixed sciatic nerve 6 h after injection, and 9 h later, the entire exposed sciatic nerve was filled with fluorescent signaling endosomes.^32^ In contrast, fluorescent signaling endosomes accumulated in different segments of the teased sciatic nerve, the teased sciatic nerve showed a relatively delayed transfer process compared to the control group mice, and the labeled AF488-CTB exhibited segmental accumulation and discontinuous status in axons. These results revealed retrograded axonal transport deficits in the PNS of *Dok6* cKO mice.

### DOK6 interacts with MAP1B to maintain axonal stability

**Fig. 6:**
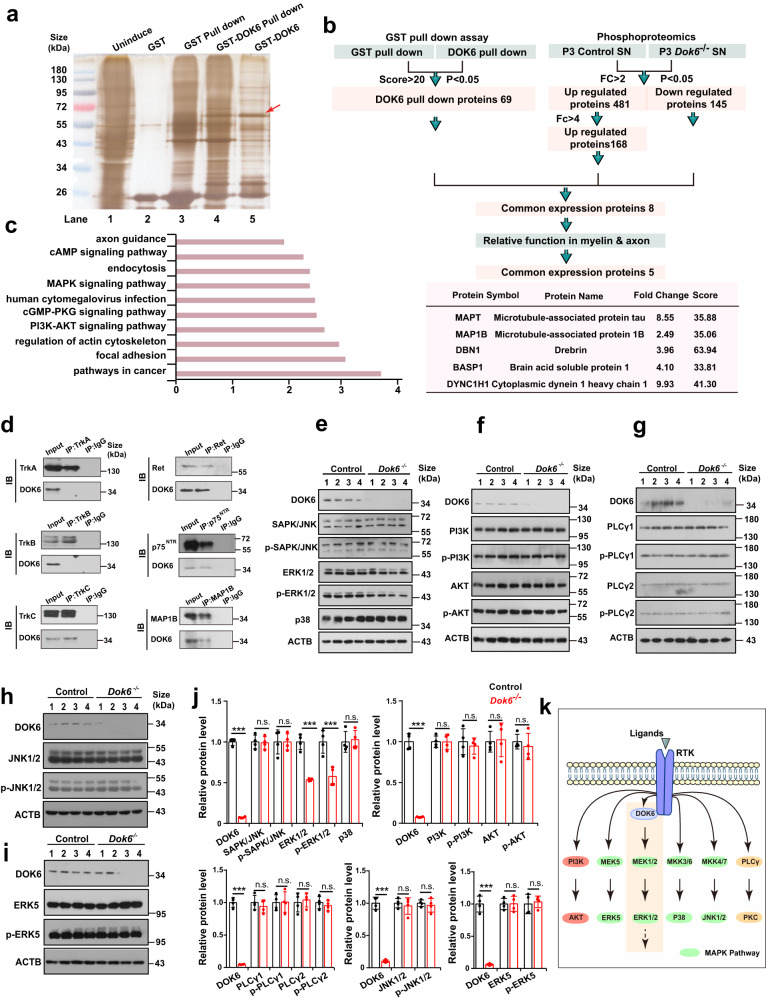
MAP1B was identified as a target of DOK6 by GST pull-down and mass spectrometry. **a** The E18.5 mouse purified brain tissue protein was incubated with the DOK6-GST fusion protein, the binding protein was retained, and then the bands were silver-stained, the red arrow indicates the location of the DOK6-GST fusion protein. Mass spectrometry analysis was performed on Lane 3, Lane 4, and Lane 5. Lane 3 and Lane 5 proteins were employed as controls to identify differential proteins. **b** The process of selecting possible interacting proteins by phosphoproteomic and GST pulldown assays. Phosphotomics mass spectrometry and GST pulldown mass spectrometry were analyzed and classified to screen candidate proteins interacting with DOK6. DOK6 potential interacting proteins, the fold change, and score were obtained based on mass spectrometry results and are listed in the lower panel. **c** The top ten GO pathways were analyzed with data from phosphoproteomics mass spectrometry assays on pulldown samples. **d** Coimmunoprecipitation assay of DOK6 with lysates from P3 mice sciatic nerve tissue. Precipitates and total lysates were subjected to Western blotting with antibodies against TrkA, TrkB, TrkC, RET, p75^NTR^, and MAP1B, revealing that DOK6 was able to bind to MAP1B. **e**–**j** Protein levels of P3 sciatic nerve protein associated with the MEK/ERK, p38, PI3K/AKT, PLCγ, JNK and MEK5/ERK5 signaling pathway assessed by Western blot in the control and *Dok6*^−/−^ groups. **k** Schematic diagram of the MEK/ERK signaling pathway in which DOK6 is involved. Data are presented as the mean ± SD, ***p < 0.001, two-tailed unpaired Student’s *t* test in Control and mutant mice

Since DOK6 acts as an adapter protein-mediated tyrosine kinase signal, to gain insight into how *Dok6* deletion leads to PNS axon degeneration, we performed a DOK6-GST pull-down assay with lysates from E18.5 brain tissue (due to the limited amount of protein in the sciatic nerve) to identify candidate DOK6-binding proteins (Fig. 6a, b). Silver staining and DOK6-specific Western blotting confirmed that purified GST-DOK6 presented as a band at the predicted molecular weight of 60 kDa. Through mass spectrometry, 69 proteins were found to potentially bind to DOK6. GO analysis of these DOK6-binding proteins revealed the enrichment of biological processes associated with axonogenesis and axonal regulation (Supplementary Fig. S5a–h, Supplementary Table S5). The RNA in situ hybridization data also confirmed that some of them were enriched in the dorsal root ganglion (Supplementary Fig. S5g).

Then, we performed phosphoproteomics in the WT and *Dok6*^−/−^ sciatic nerves at P3 to determine which signaling pathways were altered. The results showed that many molecules with altered phosphorylation levels were found in the phosphorylated proteome, and the MAPK and PI3K/AKT pathways were relatively ranked at the forefront (Fig. 6b, c). Furthermore, in combined with phosphoproteomic profiling analysis, eight proteins appeared in both experiments. Among them, five proteins have the ability to regulate axonal homeostasis or myelin formation, according to previous reports, these are microtubule-associated protein tau (MAPT), microtubule-associated protein 1B (MAP1B/MAP5), drebrin 1 (DBN1), brain acid soluble protein 1 (Basp1), and cytoplasmic dynein 1 heavy chain 1 (Dync1h1) (Fig. 6b, bottom row). Interestingly, in addition to phosphorylated Map1b, the expression of Map1b is also altered after *Dok6* knockout, which is different from other proteins (Supplementary Fig. S5j, k). This suggests that MAP1B may serve as a downstream molecule of DOK6, in addition to its binding interaction with DOK6. MAP1B is highly expressed in the mouse DRG during development and is associated with axonal degeneration, reverse axoplasmic transport, and myelin damage and remodeling.^33^ The interaction between DOK6 and MAP1B was also confirmed by coimmunoprecipitation (Co-IP) (Fig. 6d).

To identify the potential downstream pathways, we also analyzed the transcriptomic gene expression changes in WT and *Dok6* mutant sciatic nerves at P3, the stage when radial sorting is active in sciatic nerves (Supplementary Fig. S6a). 687 genes showed a significant increase, while 84 genes showed a decrease. The downregulated genes included *Dok6* and *Caps2*, a gene involved in neurotrophin release and cell survival.^34^ Strikingly, many of the myelination-related genes (e.g., *Adgrg6/Gpr126*, *Mpz*, *Erbb3*, *Lgi4*, *Egr2*) that were significantly upregulated in *Dok6* KO mice (Supplementary Fig. S6b). A subset of these genes was randomly selected for validation by quantitative real-time PCR, and the results were in line with the findings from the RNA-Seq data. (Supplementary Fig. S6c). When the differentially expressed genes (DEGs) underwent functional category analysis, they showed enrichment in “Focal adhesion” or “Neuroactive ligand-receptor interaction” pathways, as well as other signaling pathway categories in Kyoto Encyclopedia of Genes and Genomes (KEGG) analyses. Furthermore, Gene set enrichment analysis (GSEA) revealed upregulation of genes associated with the “ErbB signaling pathway” and the “Regulation of actin cytoskeleton” (Supplementary Fig. S6d, e).

Since our lab has previously revealed DOK6 is involved in Trk receptor-mediated signaling,^21^ to determine which neurotrophic factors may serve as upstream signals transduced by DOK6, we used immunoprecipitation experiments to determine whether NT receptors such as TrkA, TrkB, TrkC, Ret, and p75^NTR^ interact with DOK6 in the P3 sciatic nerve. The results show that DOK6 is capable of interacting with TrkC, Ret, p75NTR, etc., but cannot form mutual binding with TrkA and TrkB (Fig. 6d), which was subsequently confirmed in immunofluorescence experiments (Supplementary Fig. S7a). This suggests that DOK6 exhibits selectivity in mediating neurotrophin signaling.

**Fig. 7:**
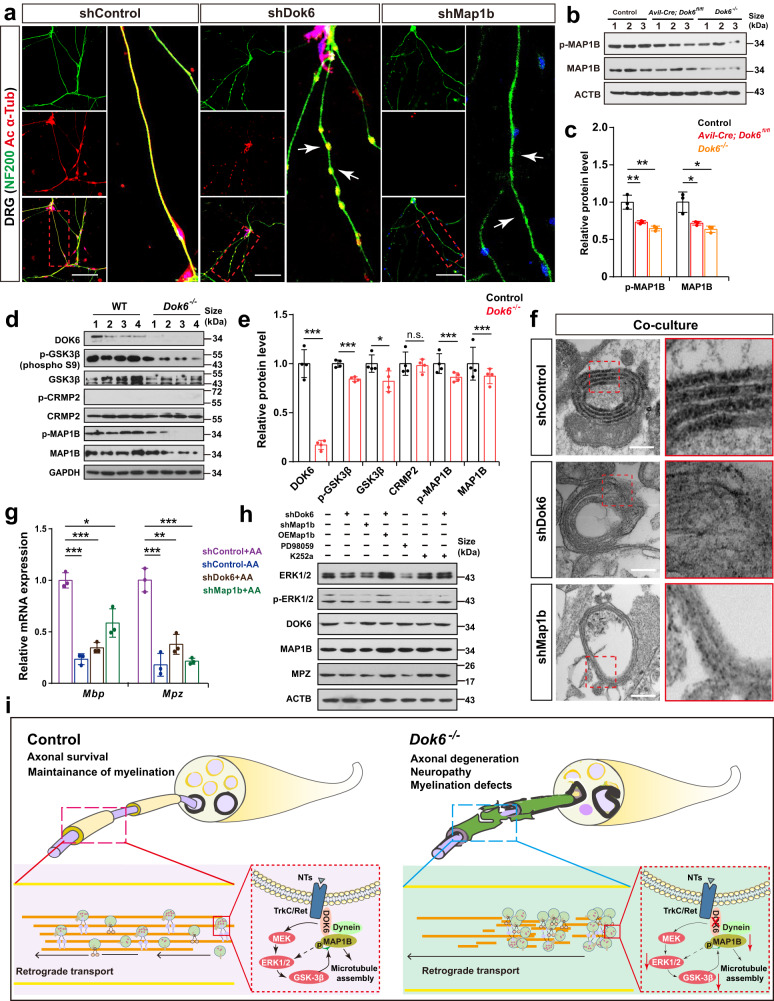
The DOK6, MAP1B and MEK/ERK signaling pathways are involved in the myelination process in the peripheral nervous system. **a** Immunocytochemistry of cultured DRG neurons after infection with sham, *Dok6-shRNA*, and *Map1b-shRNA* lentiviruses. The effects of DOK6 and MAP1B on neuronal degeneration were determined by acetylated α-tubulin (red) staining, the arrows indicate to degenerated axon showed torpedo-like structures Scale bar, 100 μm. **b**, **c** Western blot analysis of MAP1B Thr1265 phosphorylation (pMAP1B) and total MAP1B in sciatic nerves from untreated control, *Avil-Cre; Dok6*^*fl/fl*^ mice and *Dok6*^*−/−*^ mice. β-ACTIN served as a loading control. Data are presented as the mean ± SD, *p < 0.05, **p < 0.01, One-way ANOVA between different lines of mice. **d**, **e** Protein levels of P3 sciatic nerve protein associated with the GSK-3β signaling pathway, assessed by Western blot in Control and *Dok6* mutant mice sciatic nerve. Data are presented as the mean ± SD, *p < 0.05, **p < 0.01, ***p < *0.001* unpaired t test between different lines of mice. **f** Electron microscopy assay of cocultured DRG neurons and SCs infected with sham, *Dok6-*shRNA, and *Map1b-*shRNA lentiviruses. The abnormal myelin structure was exhibited in the *Dok6-*shRNA- and *Map1b-*shRNA-infected groups. Scale bar, 100 μm. **g** qRT–PCR assay for *Mbp* and *Mpz* mRNA expression in cocultured DRG neurons and SCs. *Dok6*-shRNA and *Map1b*-shRNA knockdown reduced *Mbp* and *Mpz* mRNA expression. AA refers to ascorbic acid, which is an essential component for cell myelination culture. Data are presented as the mean ± SD, *p < 0.05, **p < 0.01, ***p < 0.001, One-way ANOVA. **h** Western blot assays for DOK6, MAP1B, ERK1/2, p-ERK1/2, MPZ and ACTB in cocultured DRG neurons and SCs. K252a is an inhibitor of Trk tyrosine protein kinase, and PD98059 is a MEK/ERK pathway inhibitor. **i** Proposed schematic model of the role of DOK6 in the crosstalk between axons and glia in PNS. *Dok6* conditional deletion in neurons affected axonal degeneration and myelination caused by improper signal activation of tyrosine receptor kinase. MAP1B could interact with DOK6, and *Dok6* deletion could correspondingly reduce the phosphorylation level of MAP1B via the GSK3β signaling pathway, thus affecting the expression of ERK1/2 in the MAPK signaling pathway in neurons

Next, we investigated whether MAP1B functions downstream of DOK6 in supporting axonal maintenance. Notably, when cultured DRG neurons were infected with *Dok6*-shRNA and *Map1b*-shRNA lentivirus, the axons of the *Dok6-*shRNA group and *Map1b-*shRNA group showed discontinuous or beaded structures, as detected by immunostaining for NF200, a marker for large myelinated A fiber neurons, which was different from the littermate control group (Fig. 7a, Supplementary Fig. S7e). Moreover, the DRG neurons infected with *Dok6-shRNA* and *Map1b-shRNA*, showed a significantly decreased expression of ac-α-tub (Fig. 7a). Besides, consistent with the phosphoproteomics results, a significant decrease in MAP1B and p-MAP1B was observed in *Dok6* knockout sciatic nerves (Fig. 7b, c). These findings suggest that the function of DOK6 is related to the MAP1B in axonal degeneration.

### DOK6 affects neuronal degeneration by MEK/ERK signaling activity

We further analyzed which downstream signal is mediated by DOK6 as an adapter protein. Trk receptor signaling mainly includes MEK/ERK, PI3K/AKT, and PLC-γ.^35^ In general, the intracellular signaling pathways of receptor tyrosine kinase (RTK) have several branches: MEK1/2/ERK1/2, p38, JNK, MEK5/ERK5, and AKT.^36^ We used the sciatic nerves from P3 mice to analyze the changes in the above pathways after *Dok6* deletion. Western blot data showed that both total and phosphorylated ERK1/2 were significantly decreased in *Dok6*^−/−^ mice (Fig. 6e, j). However, elements in the PI3K/AKT pathway, PLCγ pathway, MEK5/ERK5, p38, and JNK were not altered (Fig. 6f–j). Thus, we concluded that DOK6 is involved in the MEK/ERK signaling pathway (Fig. 6k), which is important for maintaining of cell survival and regulating of peripheral myelination and axonal growth.

Next, we further explored the potential relationship between DOK6, MAP1B, and MEK/ERK1/2 signaling pathways. MAP1B is a well-known target of glycogen synthase kinase 3β (GSK3β) and is activated by GSK-3β phosphorylation. Interestingly, neurotrophin engagement with its Trk receptor enhances the activity of GSK-3β through the MAPK pathway, specifically ERK1/2 activity, then to phosphorylate MAP1B.^37^ We then investigated the expression and phosphorylation of these proteins in littermate control and *Dok6* mutant mice sciatic nerve and found that *Dok6* mutant decreased the expression and phosphorylation of GSK3β, as well as the phosphorylation of MAP1B. The expression of CRMP2 was unaffected, even the phosphorylation of CRMP2 cannot be detected (Fig. 7d, e). These results suggest that *Dok6* deletion inhibits the GSK-3β activity, which is likely to be mediated through the action of ERK1/2, thereby abolishing MAP1B phosphorylation and reducing microtubule stability. In turn, MAP1B can directly bind to DOK6 and affect the phosphorylation and expression of ERK1/2.

To further verify that the function of DOK6 is related to the MAP1B in myelination, we established an in vitro DRG neuron and SC coculture experimental system that mimics the early myelination process (Supplementary Fig. S7c). The expression of *Dok6* and *Krox20*, an SC marker, varied regularly along with the progression of myelination in the coculture system (Supplementary Fig. S7d). Immunostaining and TEM revealed degeneration in the *Dok6*-shRNA- and *Map1b-*shRNA*-*infected axons, which failed to form intact and continuous myelin sheaths. In addition, the expression of myelin protein zero (MPZ), a gene required for the proper formation and maintenance of myelin, was relatively lower in the *Dok6*-shRNA- and *Map1b-*shRNA*-*infected coculture system (Fig. 7f, Supplementary Fig. S7f). Real-time PCR analysis confirmed the decreased *Mbp* and *Mpz* mRNA expression in the coculture system upon *Dok6*-shRNA and *Map1b-*shRNA knockdown (Fig. 7g). These data indicated that MAP1B may play a similar function to DOK6 in the initiation of myelination. Notably, *Dok6*-shRNA knockdown plus *Map1b* overexpression together increased the phosphorylation level of ERK1/2 as well as the expression level of MPZ indicating that MAP1B could partially restore DOK6 function. In the *Dok6*-shRNA knockdown plus the addition of K252a, an inhibitor of tyrosine protein kinase, ERK1/2 phosphorylation levels and MPZ expression were slightly lower than those in the K252a group. PD98059, an inhibitor of the MEK/ERK pathway, efficiently blocked ERK1/2 phosphorylation in DRG neuron and SC co-culture systems and reduced MPZ expression (Fig. 7h). Taken together, these in vitro and in vivo analyses suggest that DOK6 may influence axon degeneration and then impair myelination via the MEK/ERK signaling cascade.

## Discussion

In this study, we have demonstrated that DOK6, a member of the DOK family adapter protein, is essential for axon transport, stability and myelination in the PNS (Fig. 7i). Genetic deletion of *Dok6* results in typical peripheral neuropathy symptoms such as abnormal posture and aberrant gait, nociceptive hyposensitivity, severe paw injury and deformity, peripheral myelin abnormalities, and decreased of nerve conduction velocities. These pathological changes caused by *Dok6* deletion are due to axon degeneration. Since *Avil-Cre* drives Cre-mediated recombination specifically in all peripheral sensory neurons,^30^ our data show that *Avil-Cre-*mediated *Dok6* knockout in sensory neurons displays a set of phenotypes similar to that of the *Dok6* mutant. This further indicates that the axonopathy and myelination abnormality caused by *Dok6* is mainly based on PNS sensory neurons. Mice lacking *Dok6* showed increased SMI32^+^ non-phosphorylated neurofilaments, decreased acetylation/stable α-tubulin, and impaired axonal retrograde transport in peripheral nerves. It is worth noting that the acetylation of α-tubulin promotes dynein binding to microtubules, thereby facilitating axonal retrograde transport. Decreased α-tubulin acetylation and deficits in axonal transport are considered early characteristics of peripheral neuropathy,^14^ and increasing α-tubulin acetylation and salvage axonal transport defects may provide benefits in mice with Charcot-Marie-Tooth disease (CMT), the predominant type of inherited peripheral neuropathy.^38^ Notably, the human genetic and clinical data also strongly suggest that DOK6 is closely associated with peripheral neuropathy. The single nucleotide polymorphisms of *Dok6* found to increase susceptibility to Hirschsprung’s disease, which is most commonly associated with the RET genetic mutation, indicating the relevance of *Dok6* to neurotrophic signaling.^22^ This supports the key role of *Dok6* in maintaining axon function and its possible role in the pathogenesis of peripheral neuropathy

How the specificity of functional outputs is achieved in such divergent neurotrophin signaling has always been of great interest. In this study, we found that DOK6 selectively mediates the neurotrophin signaling cascade, by selectivity binding to some neurotrophin receptor and by specifically mediating the activation of the downstream MAPK-ERK1/2 kinase pathway. First, our immunoprecipitation results showed that DOK6 can interact with TrkC and Ret but not TrkA and TrkB, and the immunofluorescence data also confirmed the co-localization of DOK6 and TrkC in DRG neurons. Meanwhile, *Dok6* deletion reduced the number of proprioceptive neurons (*TrkC*^*+*^*, Runx3*^*+*^*, PV*^*+*^) and *Ret*^*+*^ neurons in the P21 mouse DRG, but did not change the number of *TrkA*^*+*^, *CGRP*^*+*^ and *TrkB*^*+*^ DRG sensory neurons. TrkC^+^ proprioceptive neurons consist of large- and medium-diameter myelinated A-fibers (Aα and Aβ, respectively) to sense body position and movement.^24,39^ *Ret*^*+*^ neurons have two main subpopulations: small- to medium-diameter non-peptidergic nociceptors and mechanosensory neurons that sense touch.^25^ This is consistent with the behavioral changes in *Dok6* knockout mice, which are severely impaired in proprioception and experience a certain decrease in pain. Second, our phosphoproteomics and Western blot analysis in *Dok6*-deleted sciatic nerves showed that the MEK/ERK1/2 signaling pathway was significantly decreased, while other molecules (e.g., pAKT, pPLCγ1/2, pERK5, pJNK and p-p38) did not show obvious changes, suggesting that DOK6 specifically mediates the MEK/ERK signaling pathway. These results are in line with our previous findings that DOK6 can interact with the NPQY motif of TrkC receptor via its PTB domain. The tyrosine residue Tyr516, which mediates the MEK/ERK1/2 signaling pathway, is situated within the conserved NPQY motif that binds to DOK6.^21^ The reason for this is still unclear and it may be that different molecules mediate differential signal transduction at different stages or cell types.

As an adapter protein, DOK6 not only coordinates the specificity for activation of the downstream neurotrophin pathway but is also involved in the retrograde transport of NT-receptor complexes. DOK6 can directly interact with trafficking machinery proteins including MAP1B, Tau, and Dynein, and mediate their phosphorylation, to maintain axonal homeostasis and retrograde transport. Axonal transport of protein and organelle cargoes is essential for the execution and maintenance of neuron functions.^11–13^ Remarkably, several mutations in motor proteins or motor regulator genes have been identified in associated with axonal forms of hereditary peripheral neuropathies.^40,41^ For example, mutations in DYNC1H1.^42^ or RAB7A.^43^ have been reported to cause CMT. Mice with loss of microtubule-associated protein Tau or MAP1B show significant abnormalities in axon elongation,^33,44,45^ demonstrating the necessity of these MAPs in axon maintenance. We found the *Dok6* mutant decreased the expression and phosphorylated of ERK1/2, GSK3β, as well as the phosphorylation of MAP1B. Interestingly, the neurotrophin mediated MAPK-ERK1/2 pathway could phosphorylate MAP1B through activated GSK3β,^37^ and the activity of ERK1/2 regulates the cytoplasmic dynein binding to endosomes for retrograde axonal transport in response to neurotrophin.^46^ It is proposed that DOK6 can affect the activity of ERK1/2, regulate the expression and phosphorylation of MAP1B (possibly also Tau) through GSK3β to affect the stability of the cytoskeleton; and regulate cytoplasmic dynein binding to endosomes for retrograde axonal transport in response to neurotrophin. These could mechanically explain the lack of *Dok6* leading to degeneration of axons and block of axonal retrograde transport (Fig. 7i). In summary, as mentioned above, the different neurotrophic factor-receptor complexes can recruit specific adapter proteins, such as DOK6, to selectively modulate the activity of distinct targets within a network. At the same time, these adapter proteins can act as scaffolds to recruit different proteins to mediate retrograde trafficking and signaling cascades.

In *Dok6*-lacking mice, there was a significant abnormal myelination phenotype, including: (a) inhibition of radial sorting and delay of the sciatic nerve myelination at an early stage; and (b) developing neuropathy-like focal myelin abnormalities. The change in myelin sheath at the later stage may be due to the secondary effect leading to the abnormal upregulation of myelination-related gene expression, which is consistent with the sciatic nerve transcriptome results at P3. However, the expression pattern of Dok6 and the conditional knockout data suggest that the myelin abnormalities caused by *Dok6* deletion are a secondary effect of the axonal change. Mice with *Dok6* deletion in peripheral neurons by *Avil-Cre* and *Sns-Cre* showed typical peripheral myelin outfolding, axon destruction, and retrograde transport defects, whereas mice with *Dok6* deletion in SCs by *Cnp-Cre* shown no obvious changes. In the past, many studies have focused on the regulatory mechanism of axon-glia crosstalk.^3,47^ For example, it has been found neurotrophins also affect the myelination of DRG axons, that BDNF promotes myelination through the p75^NTR^ receptor, whereas NT3 inhibits myelination by acting through its TrkC receptor.^47–50^ Neuregulin 1 (Nrg1) is a crucial regulator that provides axonal signals to control Schwann cell proliferation and myelination through its ErbB2/3 receptors in SCs. Interestingly, ErbB2/3 are members of tyrosine kinase receptors, and Erk1/2 are considered to be their functional downstream cascades when activated by Nrg1.^51^ We found a significant increase in NRG1 and p-ErbB3 (ErbB3) in P3 *Dok6* knockout sciatic nerves (Supplementary Fig. S6f, g) which is consistent with our RNA-seq results (Supplementary Fig. S6). This provides some insight into the mechanism of *Dok6* mediated in axon myelination. Moreover, MAP1B is mainly expressed in neurons at high levels during development;^33^ however, our in vitro cell culture data show that knockdown of MAP1B in neurons affects the process of myelination, and overexpression of MAP1B could rescue the effect of *Dok6* knockdown on MPZ expression. This is consistent with the previously reported that mice lacking MAP1B exhibit impaired axonal myelination in the CNS and PNS.^52,53^ Humans with MAP1B loss-of-function mutations show intellectual disability and extensive white matter deficits.^54^ Franzen *et al*. found MAP1B acts as a neuronal binding partner for Myelin-associated glycoprotein (MAG), which is expressed in the periaxonal membranes of Schwann cells responsible for the maintenance of myelinated axons.^55^ Therefore, DOK6, as an important docking protein that mediates RTK signaling cascades, may be a key molecule in neurons that provides axon-glia communication in the PNS.

Taken together, this study reveals the role of DOK6 in the maintenance of peripheral nerves as a critical adapter protein that selectively facilitates part of the neurotrophin signal transduction process. These findings provide new perspectives on inherited peripheral neuropathy. Nevertheless, this study has certain limitations or raises some intriguing issues that require further investigation in the future. First, DOK6 functions as an adapter protein that can mediate the RTK pathway, although we found that DOK6 interacts with TrkC and Ret but not with TrkA and TrkB, whether it can also mediate other RTK signals and how the specificity is established needs to be further determined. Second, we found that DOK6 is expressed in the peripheral nervous system, particularly in sensory neurons. It still needs further characterization that DOK6 is expressed in which types of peripheral nerve cells, such as motor neurons (Supplementary Fig. S7b), enteric nerves, *etc*.; Third, adapter proteins can provide a docking platform to perform multiple signaling events. This study revealed that DOK6 has the capacity to bind and regulate numerous proteins in the process of prograde transport. However, the precise mechanism of the process requires further revealed; Finally, the underlying DOK6-dependent mechanism that controls the directionality of axon-glia crosstalk in the PNS remains to be elucidated. This will help us to better clarify how axonal signals recruit and maintain myelination.

## Materials and methods

### Construction of *Dok6* mutant and conditional knockout model mice

All animal experiments conducted in this study were conducted in accordance with the guidelines of the Institutional Animal Care and Use Committee of the Chinese Academy of Medical Sciences and Peking Union Medical College. Additionally, all procedures were performed in accordance with the Experimental Animal Regulations (China Science and Technology Commission Order No. 2). All mice were housed in cages with individual ventilation systems and subjected to a 12-h light/dark cycle. Except for the behavioral experiments with female mice, other experiments did not distinguish between the genders of mice. The weight of the mice ranged from 1.5 g to 25 g, with mice of different ages according to the different experimental requirements. All experiment animals were randomly allocated to different experimental groups based on their age and genotype.

*Dok6* conditional knockout mice were designed and completed by Beijing Biocytogen Co., Ltd. The *Dok6* gene has a total length of ~468 kb on chromosome 18 and has two transcripts, *Dok6*-001 (protein ID: ENSMUSP00000095103) and *Dok6*-002 (protein ID: no protein product). The knockout strategy is based on *Dok6*-001 by using the Cre/Loxp system. The mouse strains utilized in this study were bred and kept on a C57BL/6 background. The mice were obtained using the ES cells targeting strategy wherein clones exhibiting accurate recombination at both ends were selected and subsequently subjected to ES cell (on a C57BL/6 background) microinjection. The resulting offspring chimera mice were further backcrossed with the C57BL/6 wild-type mice so as to generate the mutant mice for experimentation.

A standard gene targeting procedure was used to generate the *Dok6* mutant mouse, in which a neomycin (Neo) resistance gene was flanked by exon 3 of Dok6 (Supplementary Fig. S1a). F1 heterozygous mice were first required to remove the neomycin resistance cassette. The Frt sequence is located bilaterally in the Neo cassette, which is the recognition site for FLP recombinase. Floxp (fl) heterozygous mice were obtained by crossing F1 heterozygous mice with FLP deletion mice. *EIIA-cre*, which is expressed in the early fertilized egg period, was selected to cross floxp (fl) heterozygous mice for experimental *Dok6* conventional knockout. Later, *Cnp-Cre*, *Sns-Cre*, and *Advillin-Cre* were selected to cross *Dok6*^fl/fl^ mice for *Dok6* conditional knockout.

Mice up to 7 days of age may undergo toe clipping for identification purposes, and collect the tissue in a 1.5 ml EP tube. For Mouse genome DNA extraction, 500 μL DNA Lysis Buffer and 8 μL Proteinase K were added to each tube and were fully lysed in water bath at 56 °C overnight. The EP tube was removed from the water bath and placed at room temperature for 10 min. An equal volume of phenol-chloroform mixture (25:24) was added to the tissue lysate, and centrifuged at 12,000 rpm at 4 °C for 15 min. The upper water phase of 300 μL was transferred to the new EP tube, and the same volume of isopropyl alcohol was added, and centrifuged at 12,000 rpm for 15 min at 4 °C. Leave the precipitation, add 75% ethanol to wash, centrifuge at 12,000 rpm 4 °C for 10 min, then discard the supernatant. Repeat this operation. Dry precipitation at room temperature for 5 min, adding a certain volume of ddH_2_O to dissolve.

Genotyping was conducted on DNA obtained from tail or ear biopsies using standard PCR techniques and the specified primers supplied in Supplementary Table S2, knocking out the *Dok6* gene results in the deletion of one end of the Aloxp primer and the Frt primer, the PCR assay cannot produce a fragment PCR band of the target size. The primer Aloxp was obtained by DNA identification of mice in the control group with a band size of 297 bp, and a band size of Frt is 267 bp. 3loxp primers were used for gene identification for *Dok6* knockout. The 3loxp primers located on both sides of the loxp insertion site could produce a sequence product with a length of 435 bp, but the full-length band product of control mice was 1088 bp.

### DRG dissection and culturing

Before DRG culturing, coverslips placed in 24-well plates (Corning) were treated with 20 μg/ml poly-L-lysine plus laminin for at least 12 h at 4 °C. E15.5 SD pregnant mice underwent intraperitoneal injection for anesthesia with 0.7% Amobarbital Sodium (10 ml/kg). Embryos were removed intact and placed in a 15 cm culture dish filled with normal saline. 20–24 DRGs per animal were dissected from the spinal cord regions of one mouse in the Neurobasal medium (Gibco) and collected DRGs in the L-15 medium (Gibco). Collected DRGs into the culture dish center, pipetted L-15 medium, and treated with 0.25% Trypsin and incubated at 37 °C (without CO_2_) for 45 min to 1 h. To detach the cells, 10% (vol/vol) HI-FBS was added. The cell suspension was then transferred to a sterile centrifuge tube and centrifuged at 500 × *g* for 5 min. The supernatant was removed and the cell pellet was resuspended with Neurobasal medium using fire-polished glass 0.5 Pasteur pipettes. After another centrifugation at 500×g for 5 min, the cells were resuspended in Neurobasal medium and plated or transferred to wells. The culture media, including HI-FBS, 1 mM glutamine, 10–5 M fluorodeoxyuridine, 10-5 M uridine, and 50 ng/ml NGF, were added to reach a total volume of 800 μl or 400 μl for plates or wells, respectively. The cells were maintained at 37 °C in a humidified atmosphere with 5% (v/v) CO_2_.

Then, the DRG neurons were pre-treated for 30 min in the medium with either K252a (a selective inhibitor of the tyrosine-protein kinase activity of Trk family, 100 nM, Sigma) or PD98059 (a selective inhibitor of MEK/ERK signaling, 10 μM, MCE). DMSO was added at an appropriate concentration as the solvent control for chemical reagents that required dissolution.

### DRG/Schwann cell cocultures

Myelination in DRG/Schwann cell cocultures was performed basically as described previously.^56^

### In situ hybridization and immunohistochemistry

L4 and L5 DRGs and sciatic nerves were first fixed in a solution containing 4% (wt/vol) paraformaldehyde (PFA) in DEPC H_2_O. Then were cryoprotected in a solution containing 25% (wt/vol) sucrose. After embedding in TissueTek (Sakura Finetek), the samples were sectioned into slices, thaw-mounted on Superfrost Plus slides (Erie Scientific), and stored at −80 °C.

#### In situ hybridization

RNA probes were synthesized by using gene-specific PCR primers and embryonic or adult mouse brains or spinal cords cDNA templates. In situ hybridization was performed using digoxigenin-labeled probes. The probes were then hybridized overnight at 65 °C, and the slides were treated with horseradish peroxidase anti-digoxigenin antibody (Roche). The final detection was achieved using NBT/BCIP stock solution (Roche). To estimate the neuron profile, every third section was chosen for ISH quantification, and the number of neurons positive for specific molecular markers was counted at least six independent sections were taken, with more than ten DRGs per genotype, ensuring they were nonadjacent. The primer information for the in situ hybridization probes can be found in Supplementary Table S3.

#### Immunohistochemistry

After performing antigen retrieval (10 mM citrate buffer with 0.05% Tween-20, pH 6.0, at 95 °C for 20 min) and blocking (5% goat serum plus 0.3% Triton X-100). Following that, the tissue was incubated with antibodies under different conditions. The resulting images were captured using a Zeiss LSM510 confocal microscope. To estimate the cell profile, every third section was stained with DAPI to visualize the nucleoli. The validity of the method was confirmed through several control experiments. The following primary antibodies were used: Anti-α-tubulin (CST, 3873, 1:1000), Anti-acetyl-α-tubulin (CST, 5335, 1:800), anti-TrkA (Millipore,06-574, 1:200), anti-TrkB (Santa Cruz Biotechnology, sc-377218, 1:200), anti-TrkC (R&D systems, AF1404,1:200), anti-Tuj1 (Abcam, ab78078, 1:1000), anti-S100β (Sigma, Sab4200671,1:100), anti-DOK6 (Oasisbiofarm, epitope:1624, 1:250), anti-NF200 (Abcam, ab4680,1:1000), anti-MBP (RayBiotech, Aa82-87,1: 1000), anti-MPZ (Sigma, ABN363,1:2000). Secondary antibodies were Alexa Fluor 488 (goat anti-mouse, A-11001; goat anti-rabbit, A-11008); and 594 (goat anti-rabbit, A-11012; goat anti-mouse, A-11005) (Invitrogen) with dilution: 1:1000.

### Protein extraction

Tissues were dissected from different aged mice according to the different experiments, after PBS-perfused, tissues were washed in PBS, then collected into microcentrifuge tubes and snap freezing in liquid nitrogen then stored at −80 °C. Samples were thawed on ice, and ground up in lysis buffer (Tris-NaCl-Triton-EDTA (TNTE) lysis buffer (pH = 7.4) containing 50 mM Tris-HCl, 150 mM NaCl, 0.5% Triton X-100, 1 mM EDTA, 1 mM Na_3_VO_4_, 25 mM NaF, 10 mM Na_4_P_2_O_7_·10H_2_O) with four protease inhibitors (5 μg/mL phenylmethylsulfonyl fluoride (PMSF), 0.5 μg/mL leupeptin, 0.7 μg/mL pepstatin, and 0.5 μg/mL aprotinin) and left for 30 min on ice, before clearing for 30 min at 12,000 rpm at 4 °C. Centrifuge Transfer the supernatant to the new EP tube and add about 1/5 of the supernatant volume of 6 × Protein Loading Buffer, mix well, denatured at 98 °C for 10 min in a metal bath, and store at −20 °C.

### Immunoblotting

Immunoprecipitates and protein lysates were fractionated by appropriate concentration (v/v) SDS-PAGE gels and transferred to PVDF membranes. After incubating in blocking solution (Tris-buffered saline with 0.1% (v/v) Tween 20 and 5% (w/v) non-fat dry milk) for 1 h, the membranes were then exposed to primary antibodies overnight. The next day, the membranes were washed and probed with HRP-conjugated anti-mouse or anti-rabbit secondary antibodies: Goat anti-rabbit HPR (zsbio ZB-2301), Goat anti-mouse HPR (zsbio ZB-2305), Goat anti-rat HPR (zsbio ZB-2307), Rabbit anti-goat HPR (zsbio ZB-2306), followed by detection using ECL western blotting substrate (Merck Millipore) and exposure using the Chemical Imaging System (Tanon). The following antibodies were used: anti-β-actin (Sigma, a5441), anti-DOK6 (Abcam, ab72730), anti-DOK6 (Novusbio, NBP2-16211), anti-Ret (Santa Cruz Biotechnology, sc-1290) anti-TrkA (Millipore,06-574), anti-TrkB (Santa Cruz Biotechnology, sc-377218), anti-TrkC (R&D systems, AF1404), anti-MAP1B (Santa Cruz Biotechnology, sc-136472), anti-p-MAP1B (Novus Biologicals, NBP1-42827), anti-p75^NTR^ (Abcam, ab52987), anti-p-MAPK kit (CST, 9910T), anti-MAPK kit (CST, 9926T), anti-p-ERK1/2 kit (CST, 9911T), anti- AKT kit (CST, 9916T), anti- PI3K kit (CST, 9655T), anti- AKT (CST, 9272S) anti- PLCγ kit (CST, 3860T), anti- ERK5 (CST, 3372), anti- p-ERK5 (CST, 3371), anti- JNK1/JNK2 (Abcam, ab4821). Anti-α-tubulin (CST, 3873), Anti-acetyl-α-tubulin (CST, 5335), anti-MAPT (Santa Cruz Biotechnology, sc-166062), anti-DBN1 (Abcam, ab60933), anti-BASP1 (Abcam, ab214322), Anti-p-GSK3β (CST, 9336), Anti-GSK3β (CST, 12456), Anti-p-CRMP2 (Abcam, ab62478), Anti-CRMP2 (CST, 9393), Anti-NRG1 (Abcam, ab180808), Anti-p-ErbB3 (CST, 4791), Anti- ErbB3 (Abcam, ab32121).

### GST pull-down assays

Clone the *Dok6* cDNA into the GST expression vector pGEX-6p-1, then transform constructs into the *E. coli* strain BL21 (DE3), subsequently induced with1 mM IPTG and incubate with 30°C while shaking at 220 rpm for 6 h. The recombinant GST fusion proteins were purified using Glutathione Sepharose Resin (TaKaRa). After the immobilization of the bait onto the prepared resin, the E18.5 mice forebrain lysates were incubated with GST-Full-length-DOK6 (GST-DOK6) or GST alone (negative control) coupled to glutathione Sepharose beads overnight at 4 °C. After extensive washing of the pelleted glutathione Sepharose beads, collect the beads, add 1XPBS for re-suspension. Analyze the results using SDS-PAGE, the precipitated material was analyzed by silver staining.

### Immunoprecipitations

Sciatic nerve protein collected from P3 mice was lysed, and protein was subjected to immunoprecipitation using TrkA, TrkB, TrkC, RET, p75^NTR^, and MAP1B specific antibodies or isotype control antibodies with protein G-conjugated Sepharose beads while rotating overnight at 4 °C. After incubation, the immunoprecipitates were subjected to four washes with lysis buffer and then eluted in 20 μl of 6× loading buffer at 98 °C.The eluted samples were subsequently analyzed by western blotting using their corresponding anti-DOK6 antibodies.

### Lentiviral and adenoviral production and infection

Lentiviruses for overexpression and knockdown of *Dok6* and *Map1b* were packaged by Shanghai Genechem Co., Ltd. Infected cells were treated with puromycin (2 μg/ml) for 14 days to select for successfully infected cells. GFP expression was observed to determine the efficiency of cell infection.

### Quantitative reverse transcription PCR (qRT–PCR)

Total RNA was isolated using TRIzol reagent (Invitrogen), and cDNA was synthesized using TransScript First-Strand cDNA Synthesis SuperMix (TransGen). qRT-PCR assays were conducted with SYBR Premix EX Taq (TaKaRa) and the IQ5 sequence detection system (Applied Biosystems) was used to record the signals. Each experiment included triplicate samples and the assay was repeated three times. The primer sequences can be found in the methods section, with Gapdh serving as the internal control. Supplementary Table S4 provides the qRT-PCR primer information.

### Retrograde tracer injection

Preparation of 0.5% (wt/vol) Alexa Fluor 488-conjugated cholera toxin-B (AF488- CTB, Thermo Fisher, C34775) solution involved dissolving 100 μg of solid in 20 μl of phosphate buffer. In a clean and well-ventilated environment, the fur was removed from the skin that covered the gastrocnemius and anterior tibialis muscles of the lower limb on one side of the mouse, the clipped hair was removed using surgical tape, the right leg was extended, the top of the hind paw was tapped, and straight forceps were used to gently hold the skin in place. Two small incisions were made, one above the gastrocnemius muscle and one below the anterior tibialis muscle. The solution containing the fluorescent probe was placed in a microsyringe, and 2 µl was slowly injected into the muscles. It was ensured that the eye of the needle could be seen within the muscle before the injection. The injection into the lateral head of the gastrocnemius was performed at a steep angle relative to the table surface, with a much shallower, almost horizontal approach, angle (10–20°) for the tibialis anterior. Once the injection was finished, the microsyringe in the muscles was halted for 3–5 s and then slowly removed.

### Nerve isolation and teasing of nerve fibers

Sciatic nerves were obtained from 6-week-old mice at least 4 h after AF488-CTB injection, and the surviving mice were anesthetized with 0.7% amobarbital (10 ml/kg, i.p.) Then, sciatic nerves were isolated from the mice and fixed in 4% ice-cold paraformaldehyde in 0.1 M phosphate buffer pH 7.4 and ddH_2_O for 30 min, rinsed three times with 0.1 M PBS, and stored at 4 °C until teased; teased sciatic nerve fibers were mounted on slides and stored at room temperature for drying up to 8 h before analysis using an Olympus confocal microscope.

### Electron microscopy

The perfusion-fixed tissues from transcardially perfused mice were fixed in a solution containing 4% paraformaldehyde (wt/vol) then postfixed in a solution containing 4% paraformaldehyde and 2.5% glutaraldehyde (wt/vol). The tissues from control and *Dok6* mutant mice were prepared together. Digital images were captured at original magnifications of ×5000 and ×8000. The g-ratio was measured on electron micrographs of randomly chosen axons per animal. The number and percentage of myelinated axons were counted on complete semithin cross-sections of sciatic nerves. The percentage of abnormal myelinated axons was quantified by counting all fibers on randomly selected electron micrographs at the same magnification per animal.

### g-ratio statistical analysis

Statistical analysis g-ratio, calculated as the ratio of axon diameter to fiber diameter, was performed as previously described.^57^ For quantification, sciatic nerve axons were manually counted using ImageJ software. The scale bar corresponding to the magnification used in each micrograph was used to calibrate the images. Randomly selected fibers were used to measure axons without myelin and the fiber regions with myelin, excluding those surrounded by a Schmidt Lanterman incisure. A minimum of 100 nerve fibers were analyzed per animal.

### Nerve conduction velocity measurement

Conduction velocity measurements were performed on P60 mice under anesthesia (0.7% amobarbital 10 ml/kg). The sciatic nerve tissue was removed from each animal, and then by stimulating one end of the motor nerve, the impulse was transmitted along the nerve trunk and recorded at the other end of the recording electrodes. The distance between the stimulation electrodes and recording electrodes was fixed, and compound action potentials (CAPs) were recorded with a BL-420N Biological Signal Acquisition and Analysis System (Chengdu, China). The calculation of nerve conduction velocities involved determining the distance between the stimulation and recording electrodes and measuring the latency difference between the successive stimulation-induced CAPs.

### Behavioral tests

Mice were maintained on a 12-h light/dark cycle and provided with ad libitum access to food and water. A 1-h habituation period was given to the mice before the start of the experiments. All behavioral experiments were conducted blindly.

#### The grip strength test

The forelimb grip strength test is a frequently employed technique where an examiner pulls the tail of a rodent horizontally while it grips a bar connected to a monitoring device. The maximum value recorded during this test represents the forelimb grip strength.

#### Coat hanger test

The experiment can detect comprehensive animal muscle motion ability, and objectively reflect the central nervous system of motor coordination ability and control ability. Use hangers as hanging tools, when the mouse catches its front paws hanging objects, timing starts, record its downtime, the time of climbing on the coat hanger, and the incubation period of falling were recorded.

#### Balance beam walking

Motor and balance coordination were evaluated by assessing the ability of mice to cross the balance beam laterally to reach a closed safety platform. The mice were placed on a balance beam with a length of 1 m and a width of 1 cm, and the time mice took to walk from one end of the balance beam to the other end (from the beginning of one end of the balance beam to the time when all their limbs entered the other end of the balance beam) was recorded to evaluate the balance ability.

#### Hot plate

Mice were initially placed on a plate set at 48 °C at initially. The latency to the onset of hindpaw licking or shaking was measured. Each mouse was tested twice with a resting period of 5 min between trials. After a resting period of at least 1 h, the experiment was repeated at 50 °C and 52 °C. A cut-off of 60 s was applied to avoid tissue damage.

#### Rotarod test

Mice were subjected to the rotarod test using the LSI Letica Scientific Instruments apparatus. The rotarod was set to gradually accelerate from 4 to 40 rpm over a 5-min period, and the time taken for the mice to fall off was recorded. The mice underwent training and testing for 4 consecutive days, with each day consisting of three separate tests, each separated by at least a 5-min resting period.

#### Treadmill exercise

Each mouse was familiarized with the treadmill apparatus (Model T306, Diagnostic & Research Instruments Co., Taoyuan, Taiwan). The mice were then made to exercise at a speed of 15 m/min for a duration of 10 min, while an electric shock grid on the rear barrier was used to motivate them to run. The distance covered by the mice during this exercise was measured.

### RNA-seq and data analysis

The sequencing process includes the detection of total RNA samples, construction of libraries, inspection of libraries, and sequencing. The RNA-seq libraries were prepared using NEBNext® Ultra™ Directional RNA Library Prep Kit for Illumina® and then sequenced by Illumina NovaSeq 6000 sequencer. Once the database meets the qualifying criteria, sequencing is carried out after pooling according to the requirements of effective concentration and target data quantity. After obtaining clean data, reads can be compared, and then performed gene expression quantification, differential expression analysis, and functional enrichment analysis using R language (version 4.2.1, http://www.rproject.org). Differential expression analysis of two conditions was performed using the DESeq2R package (version 1.38.3). Genes with an adjusted *P* value (padj) <0.05 and log2FoldChange > 2 found by DESeq2 were assigned as differentially expressed. The clusterProfiler package (4.6.2) was utilized for conducting the GO, KEGG, and GSEA analyses. Moreover, each genotype sample underwent three biological replicates. The sequencing depth of RNA-seq in this study is within the range of 45-50 M mapped reads per sample.

### Statistical analysis

All experiments, except for Western blot and quantitative reverse transcription PCR analyses, were conducted in a single-blinded manner. GraphPad Prism 6.0 software was used for generating plots and performing statistical analyses. Data were analyzed using a two-tailed unpaired Student’s *t* test or a one-way ANOVA if the data followed a normal distribution. If the data did not pass the normality test, Mann-Whitney *U* tests, or Kruskal-Wallis tests with Dunn’s multiple comparison tests were utilized. *p* values below 0.05 were considered significant (* < 0.05; ** < 0.01; *** < 0.001). The results are presented as the mean ± SD. Molecular and biochemical analyses were performed with a minimum of three biological replicates for each condition.

### Supplementary information

Dok6-Supplementary Materials

Supplemental Video 1

## Data Availability

The RNA-seq data have been deposited in NCBI’s Gene Expression Omnibus and are accessible through GEO Series accession number GSE249502. The data that support the findings of this study will be made available to the scientific community upon reasonable request.

## References

[1] Lemmon MA, Schlessinger J (2010). Cell signaling by receptor tyrosine kinases. Cell.

[2] Clark JF, Soriano PM (2022). Pulling back the curtain: the hidden functions of receptor tyrosine kinases in development. Curr. Top. Dev. Biol..

[3] Pease-Raissi SE, Chan JR (2021). Building a (w)rapport between neurons and oligodendroglia: reciprocal interactions underlying adaptive myelination. Neuron.

[4] Fantauzzo KA, Soriano P (2015). Receptor tyrosine kinase signaling: regulating neural crest development one phosphate at a time. Curr. Top. Dev. Biol..

[5] Zweifel LS, Kuruvilla R, Ginty DD (2005). Functions and mechanisms of retrograde neurotrophin signalling. Nat. Rev. Neurosci..

[6] Scott-Solomon E, Kuruvilla R (2018). Mechanisms of neurotrophin trafficking via Trk receptors. Mol. Cell Neurosci..

[7] Harrington AW, Ginty DD (2013). Long-distance retrograde neurotrophic factor signalling in neurons. Nat. Rev. Neurosci..

[8] Chowdary PD, Che DL, Cui B (2012). Neurotrophin signaling via long-distance axonal transport. Annu. Rev. Phys. Chem..

[9] Indo Y (1996). Mutations in the TRKA/NGF receptor gene in patients with congenital insensitivity to pain with anhidrosis. Nat. Genet..

[10] Mardy S (1999). Congenital insensitivity to pain with anhidrosis: novel mutations in the TRKA (NTRK1) gene encoding a high-affinity receptor for nerve growth factor. Am. J. Hum. Genet..

[11] Beijer D, Sisto A, Van Lent J, Baets J, Timmerman V (2019). Defects in Axonal Transport in Inherited Neuropathies. J. Neuromuscul. Dis..

[12] Sleigh JN, Rossor AM, Fellows AD, Tosolini AP, Schiavo G (2019). Axonal transport and neurological disease. Nat. Rev. Neurol..

[13] Perlson E, Maday S, Fu MM, Moughamian AJ, Holzbaur EL (2010). Retrograde axonal transport: pathways to cell death?. Trends Neurosci..

[14] Mo Z (2018). Aberrant GlyRS-HDAC6 interaction linked to axonal transport deficits in Charcot-Marie-Tooth neuropathy. Nat. Commun..

[15] Brummer T, Schmitz-Peiffer C, Daly RJ (2010). Docking proteins. FEBS J..

[16] Borowicz P, Chan H, Hauge A, Spurkland A (2020). Adaptor proteins: flexible and dynamic modulators of immune cell signalling. Scand. J. Immunol..

[17] Wagner MJ, Stacey MM, Liu BA, Pawson T (2013). Molecular mechanisms of SH2- and PTB-domain-containing proteins in receptor tyrosine kinase signaling. Cold Spring Harb. Perspect. Biol..

[18] Pawson T (2007). Dynamic control of signaling by modular adaptor proteins. Curr. Opin. Cell Biol..

[19] Crowder RJ, Enomoto H, Yang M, Johnson EM, Milbrandt J (2004). Dok-6, a novel p62 Dok family member, promotes Ret-mediated neurite outgrowth. J. Biol. Chem..

[20] Mashima R, Hishida Y, Tezuka T, Yamanashi Y (2009). The roles of Dok family adapters in immunoreceptor signaling. Immunol. Rev..

[21] Li W (2010). Downstream of tyrosine kinase/docking protein 6, as a novel substrate of tropomyosin-related kinase C receptor, is involved in neurotrophin 3-mediated neurite outgrowth in mouse cortex neurons. BMC Biol..

[22] Lan C (2021). Association between ABHD1 and DOK6 polymorphisms and susceptibility to Hirschsprung disease in Southern Chinese children. J. Cell Mol. Med..

[23] Lakso M (1996). Efficient in vivo manipulation of mouse genomic sequences at the zygote stage. Proc. Natl Acad. Sci. USA.

[24] Lallemend F, Ernfors P (2012). Molecular interactions underlying the specification of sensory neurons. Trends Neurosci..

[25] Luo W (2007). A hierarchical NGF signaling cascade controls Ret-dependent and Ret-independent events during development of nonpeptidergic DRG neurons. Neuron.

[26] Feltri ML, Poitelon Y, Previtali SC (2015). How Schwann cells sort axons. Neuroscientist.

[27] Rossor AM, Polke JM, Houlden H, Reilly MM (2013). Clinical implications of genetic advances in Charcot-Marie-Tooth disease. Nat. Rev. Neurol..

[28] Lappe-Siefke C (2003). Disruption of Cnp1 uncouples oligodendroglial functions in axonal support and myelination. Nat. Genet..

[29] Agarwal N, Offermanns S, Kuner R (2004). Conditional gene deletion in primary nociceptive neurons of trigeminal ganglia and dorsal root ganglia. Genesis.

[30] Zurborg S (2011). Generation and characterization of an Advillin-Cre driver mouse line. Mol. Pain..

[31] Petzold A (2005). Neurofilament phosphoforms: surrogate markers for axonal injury, degeneration and loss. J. Neurol. Sci..

[32] Sleigh JN, Tosolini AP, Schiavo G (2020). In vivo imaging of anterograde and retrograde axonal transport in rodent peripheral nerves. Methods Mol. Biol..

[33] Villarroel-Campos D, Gonzalez-Billault C (2014). The MAP1B case: an old MAP that is new again. Dev. Neurobiol..

[34] Sadakata T (2004). The secretory granule-associated protein CAPS2 regulates neurotrophin release and cell survival. J. Neurosci..

[35] Arevalo JC, Wu SH (2006). Neurotrophin signaling: many exciting surprises!. Cell Mol. Life Sci..

[36] Dinsmore CJ, Soriano P (2018). MAPK and PI3K signaling: at the crossroads of neural crest development. Dev. Biol..

[37] Goold RG, Gordon-Weeks PR (2005). The MAP kinase pathway is upstream of the activation of GSK3beta that enables it to phosphorylate MAP1B and contributes to the stimulation of axon growth. Mol. Cell Neurosci..

[38] d’Ydewalle C (2011). HDAC6 inhibitors reverse axonal loss in a mouse model of mutant HSPB1-induced Charcot-Marie-Tooth disease. Nat. Med..

[39] Meltzer S, Santiago C, Sharma N, Ginty DD (2021). The cellular and molecular basis of somatosensory neuron development. Neuron.

[40] McCray BA, Scherer SS (2021). Axonal Charcot-Marie-Tooth disease: from common pathogenic mechanisms to emerging treatment opportunities. Neurotherapeutics.

[41] Klein CJCharcot-Marie-Tooth (2020). Disease and Other Hereditary Neuropathies. Contin.

[42] Weedon MN (2011). Exome sequencing identifies a DYNC1H1 mutation in a large pedigree with dominant axonal Charcot-Marie-Tooth disease. Am. J. Hum. Genet..

[43] Verhoeven K (2003). Mutations in the small GTP-ase late endosomal protein RAB7 cause Charcot-Marie-Tooth type 2B neuropathy. Am. J. Hum. Genet..

[44] Takei Y, Teng J, Harada A, Hirokawa N (2000). Defects in axonal elongation and neuronal migration in mice with disrupted tau and map1b genes. J. Cell Biol..

[45] Utreras E (2008). Microtubule-associated protein 1B interaction with tubulin tyrosine ligase contributes to the control of microtubule tyrosination. Dev. Neurosci..

[46] Mitchell DJ (2012). Trk activation of the ERK1/2 kinase pathway stimulates intermediate chain phosphorylation and recruits cytoplasmic dynein to signaling endosomes for retrograde axonal transport. J. Neurosci..

[47] Nave KA, Trapp BD (2008). Axon-glial signaling and the glial support of axon function. Annu. Rev. Neurosci..

[48] Cosgaya JM, Chan JR, Shooter EM (2002). The neurotrophin receptor p75NTR as a positive modulator of myelination. Science.

[49] Woolley AG (2008). Developmental loss of NT-3 in vivo results in reduced levels of myelin-specific proteins, a reduced extent of myelination and increased apoptosis of Schwann cells. Glia.

[50] Chan JR, Cosgaya JM, Wu YJ, Shooter EM (2001). Neurotrophins are key mediators of the myelination program in the peripheral nervous system. Proc. Natl Acad. Sci. USA.

[51] Birchmeier C, Bennett DL (2016). Neuregulin/ErbB signaling in developmental myelin formation and nerve repair. Curr. Top. Dev. Biol..

[52] Meixner A (2000). MAP1B is required for axon guidance and Is involved in the development of the central and peripheral nervous system. J. Cell Biol..

[53] Takei Y (1997). Delayed development of nervous system in mice homozygous for disrupted microtubule-associated protein 1B (MAP1B) gene. J. Cell Biol..

[54] Walters GB (2018). MAP1B mutations cause intellectual disability and extensive white matter deficit. Nat. Commun..

[55] Franzen R (2001). Microtubule-associated protein 1B: a neuronal binding partner for myelin-associated glycoprotein. J. Cell Biol..

[56] Paivalainen S (2008). Myelination in mouse dorsal root ganglion/Schwann cell cocultures. Mol. Cell Neurosci..

[57] Kaiser T (2021). MyelTracer: a semi-automated software for myelin g-ratio quantification. eNeuro.

