# Supplementary material for: Docking protein 6 (DOK6) selectively docks the neurotrophic signaling transduction to restrain peripheral neuropathy
Source: Signal Transduct Target Ther. 2024 Feb 14;9:32. doi: 10.1038/s41392-024-01742-2 (PMC10864363; doi:10.1038/s41392-024-01742-2)
Supplement: Supplementary file 1 — Dok6-Supplementary Materials [file 41392_2024_1742_MOESM1_ESM.docx]

Supplementary Materials for

**DOK6 Selectively Docks the Neurotrophic Signaling Transduction to Restrain Peripheral Neuropathy**

Yan Guo^1,7^, Pan Xiang^1,7^, Xiaojiao Sun^1^, Wei Liu^1^, Jiafeng Zhou^1^, Bin Yin^1,2^, Lin Hou^1,2^, Boqin Qiang^1,2^, Huiliang Li^6^, Pengcheng Shu^1,2,5*^, Xiaozhong Peng^1,3,4*^

Correspondence to: pengxiaozhong@pumc.edu.cn;
pengcheng_shu@ibms.pumc.edu.cn

**This PDF file includes:**Materials and Methods
Supplementary Text
Figures. S1 to S7
Tables S1 to S5

**Other Supplementary Materials for this manuscript include the following:**Movies S1


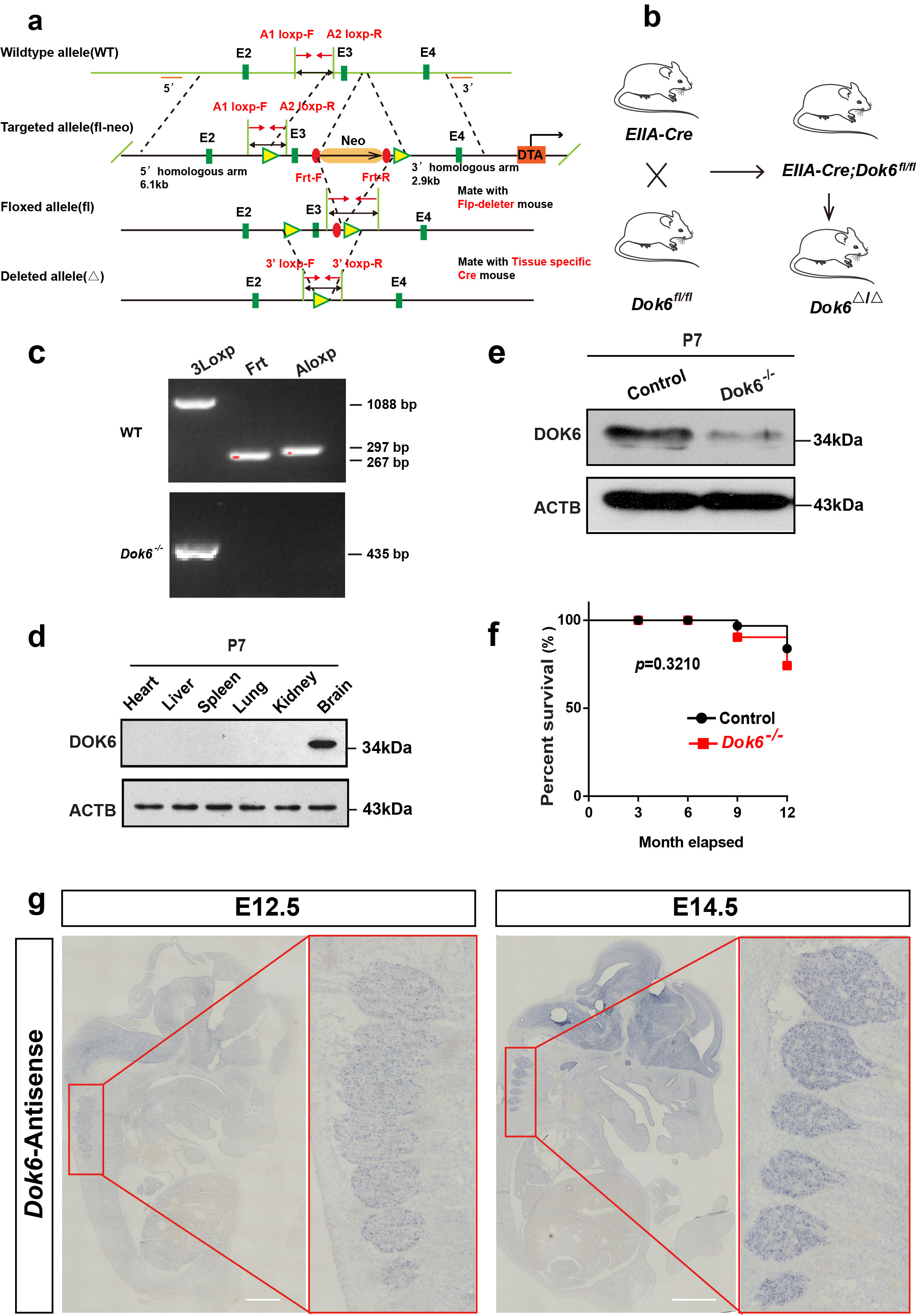


**Figure S1.** **Identification of *Dok6*^-/-^ mice. (a)** Targeted disruption of the mouse *Dok6* gene. The structures of the *Dok6* wild-type allele, the targeted allele, the floxed allele after Flp recombination, and the deleted allele after Cre recombination are indicated as dotted lines. The *Dok6* gene consists of 8 exons, and the targeting vector contained loxP sites (yellow triangles) within introns 2 and 3. **(b)** Mating program of *Dok6* knockout mice. **(c)** The target designed primers of knockout mice were used to conduct DNA level detection. The control group mice DNA identification produce primers Aloxp with 297 bp and Frt with 267 bp bands in size respectively. 3loxp primers were used for *Dok6* mutant gene identification, producing 1088 bp bands in control group and 435 bp bands in *Dok6* mutants. **(d)** Western blots with protein lysates from different tissues of control group mice showed that DOK6 is expressed in the brain. β-actin (ACTB) serves as a loading control. **(e)** Western blot analysis of protein lysates from the brains of control group mice and *Dok6^-/-^* mice. DOK6 expression was not detected in *Dok6^-/-^* brain. **(f)** Survival proportions of control (n = 30) and *Dok6^-/-^* (n = 30) group mice. Mantel-Cox statistical test, *Dok6* mutants vs Control group. **(g)** *Dok6* RNA *in situ* hybridization of sagittal sections from mouse embryonic tissues. *Dok6* was expressed in the DRG at E12.5 and E14.5. Scale bar, 1mm.


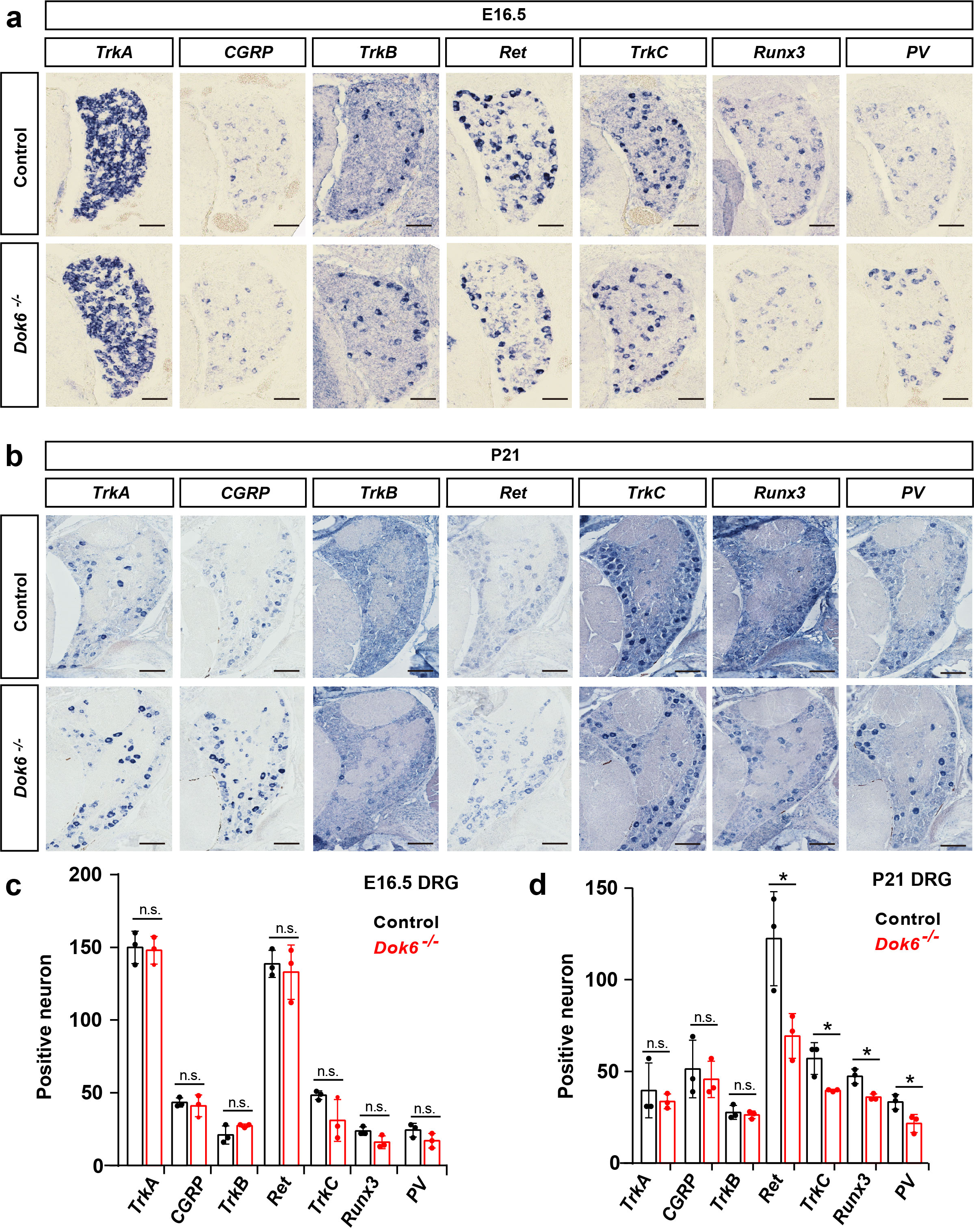


**Figure S2.** **The development of most DRG sensory neurons was not affected in *Dok6^-/-^* mice**. **(a, b)** *In situ* hybridizations were performed on transverse lumbar sections from control and *Dok6*^-/-^ mice at E16.5 and P21. The number of nociceptive (*TrkA*^+^, *CGRP*^+^), mechanoreceptive (*TrkB*^+^ or *Ret*^+^) and proprioceptive (*TrkC^+^*, *Runx3^+^*, *PV^+^*) sensory neurons was analyzed. Note that embryonic *TrkA*^+^ neurons can give rise to postnatal *TrkA*^+^ peptidergic and *Ret^+^* non-peptidergic neurons. Scale bar, 100 μm. **(c, d)** Quantification of *TrkA*^+^, *CGRP*^+^, *TrkB*^+^, *Ret*^+^, *TrkC*^+^, *TrkB*^+^, *Runx3*^+^ and *PV*^+^ neurons at E16.5 and P21 in the DRG. No significant changes were observed in most sensory neurons were observed in the *Dok6* DRG compared to the control group DRG, except for *TrkA*^+^ neurons and *TrkC*^+^ neurons at E16.5 and *CGRP*^+^ neurons and *TrkC*^+^ neurons at P21. (n = 3 per genotype). Data are presented as the mean ± SD, **p < 0.05,* unpaired t-test between control and mutant mice.


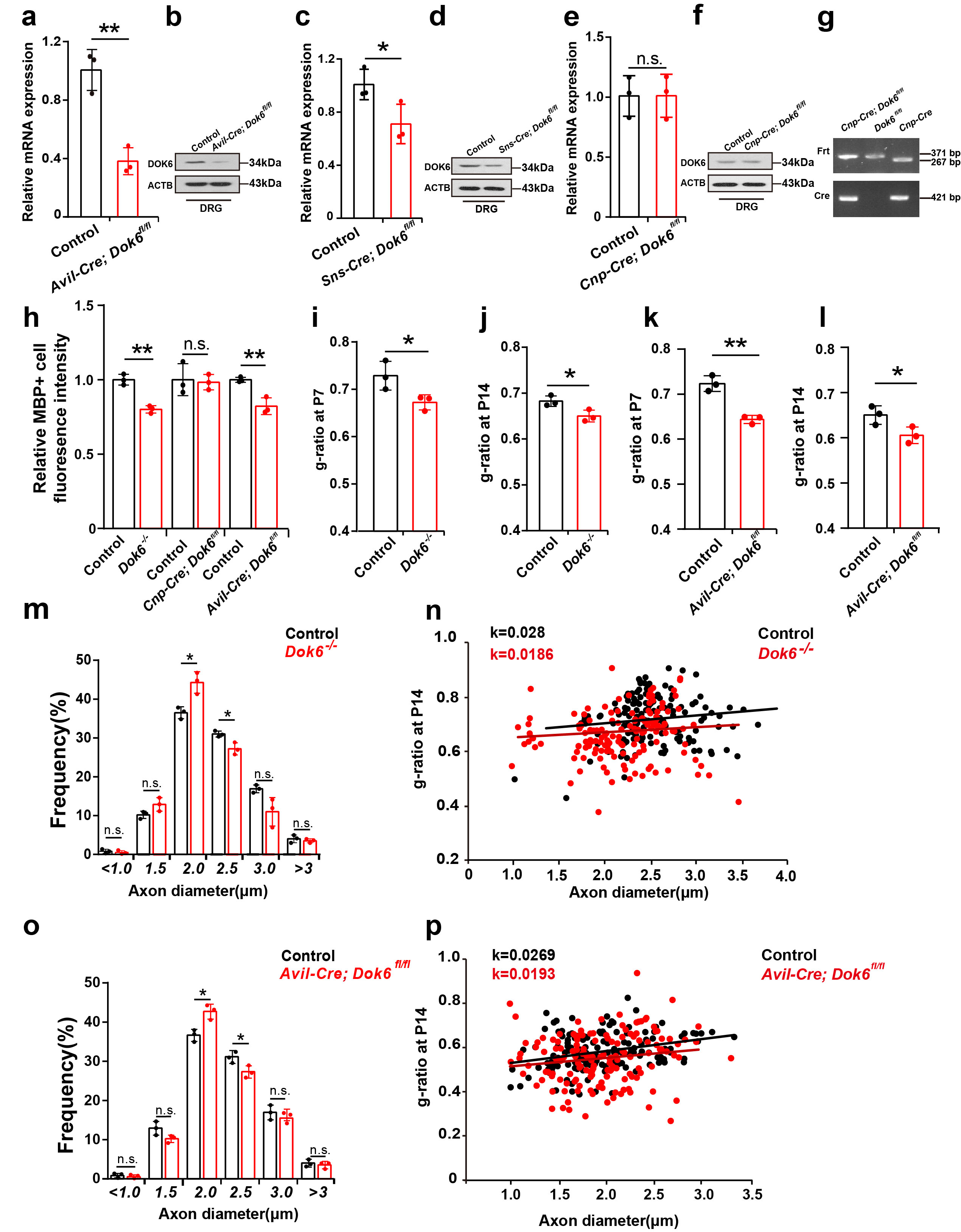


**Figure S3. Mice lacking *Dok6* in neurons rather than in SCs showed abnormal peripheral myelin. (a, c and e)** qRT–PCR of *Dok6* mRNA from the DRGs of *Avil-Cre; Dok6^fl/fl^* (A), *Sns-Cre; Dok6^fl/fl^* (C), *Cnp-Cre; Dok6^fl/fl^* (E) and their respective littermate control mice at P7. **(b, d and f)** DOK6 protein expression was detected by Western blot in the *Avil-Cre; Dok6^fl/fl^* (B), *Sns-Cre; Dok6^fl/fl^* (D), *Cnp-Cre; Dok6^fl/fl^* (F) and their respective littermate and littermate control DRG at P7. **(g)** PCR analysis of tail DNA for genotyping *Cnp-Cre; Dok6^fl/fl^* mice*.* Frt refers to primer Frt-F/Frt-R. **(h)** Relative fluorescence intensity of MBP- positive cell from sciatic nerve of P7 control, *Dok6* mutant, *Cnp-Cre; Dok6 ^fl/fl^* and *Avil-Cre; Dok6^fl/fl^* mice (n = 3 per genotype). **(i-l)** Myelin sheath thickness was quantified using modified g-ratio analysis of the sciatic nerve of control, *Dok6 ^-/-^* and *Avil-Cre*; *Dok6 ^fl/fl^* mice at P7 and P14 (n = 3 animals per genotype). **(m，o)** The distribution of axons with different diameters of the sciatic nerve in control, *Dok6 ^-/-^* and *Avil-Cre*; *Dok6 ^fl/fl^* mice at P14. **(n, p)** The relationship between the g-ratio and sciatic nerve axon diameter in control, *Dok6 ^-/-^* and *Avil-Cre*; *Dok6 ^fl/fl^* mice at P14. The thickness of the myelin sheaths of axon of different diameters was measured. Data are presented as the mean ± SD, **p < 0.05, **p < 0.01,* two-tailed unpaired Student’s t test.


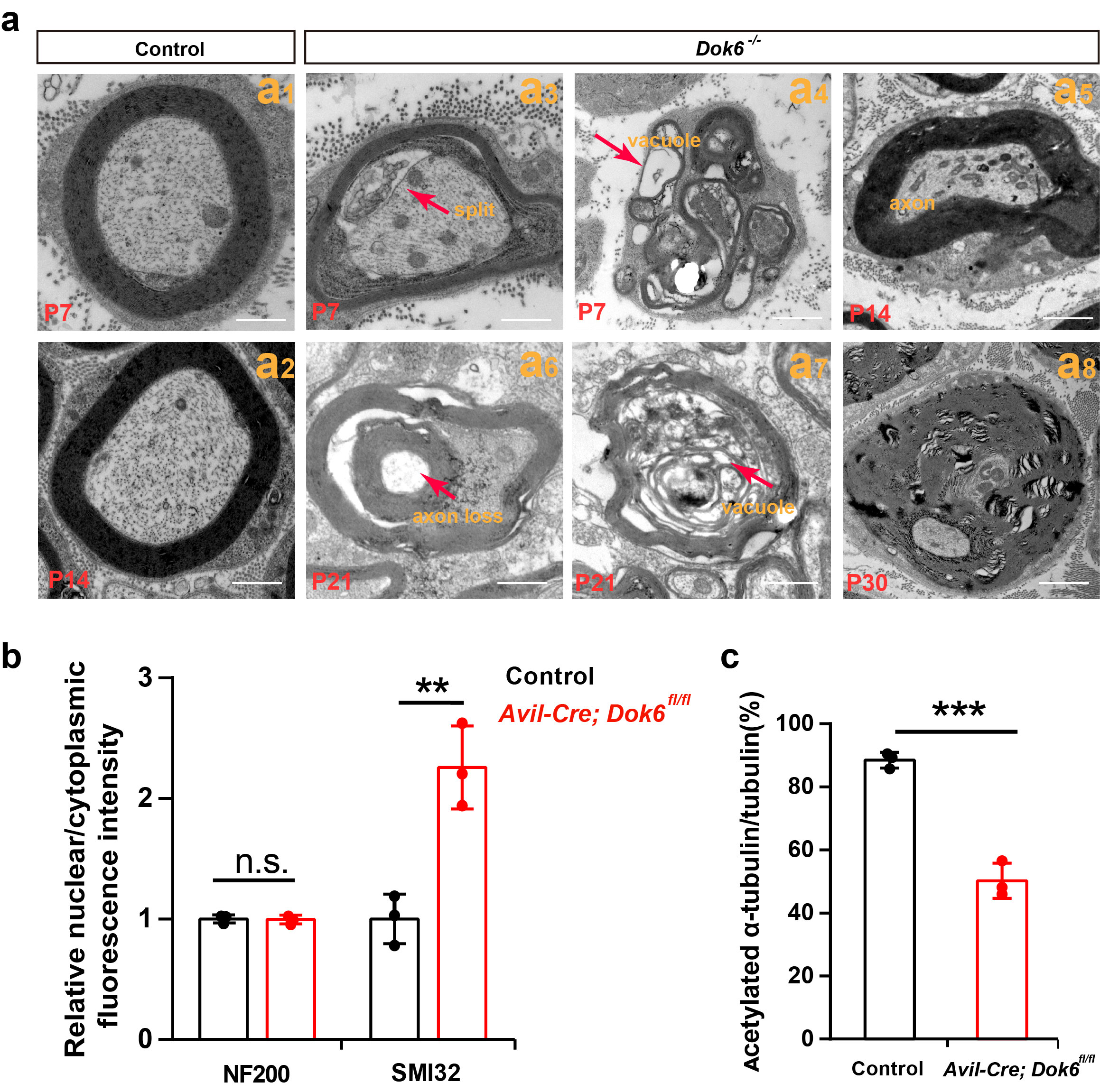


**Figure S4. Mice lacking *Dok6* exhibited axonal degeneration. (a)** Ultrastructure of the sciatic nerve observed by TEM in control and *Dok6^-/-^* mice. Axon pathological alterations were present in *Dok6^-/-^* mice. The normal myelinated axons of nerve fibers have a pale axoplasm with neurofilaments and microtubules homogeneously dispersed, as well as occasional profiles of mitochondria and smooth endoplasmic reticulum (a_1_, a_2_), In the sciatic nerve of *Dok6* mutant mice, the axon is detached from the myelin (a_3_), the axoplasmic contents appear to be vacuolated (a_4_), contains neurofilaments surrounded by an accumulation of lysosomes and mitochondria or other membrane bound organelles (a_5_), some structures that have the appearance of myelin pieces and are characterized by axon loss and myelin lamellae dense cytoplasm (a_6_, a_7_), the much smaller axon within the over expanded myelin sheath has degenerated (a_8_). Scale bar, 500 nm. **(b)** Relative nuclear /cytoplasmic fluorescence intensity of NF200 and SMI32 in the sciatic nerve of P7 control and *Avil-Cre;* *Dok6 ^fl/fl^* mice (n = 3 per genotype). **(c)** The percentage of Acetylated α-tubulin/ total tubulin in P7 control and *Avil-Cre*; *Dok6 ^fl/fl^* mouse sciatic nerve (n = 3 per genotype). Data are presented as the mean ± SD, ***p < 0.01,* ****p< 0.001*, unpaired t test.


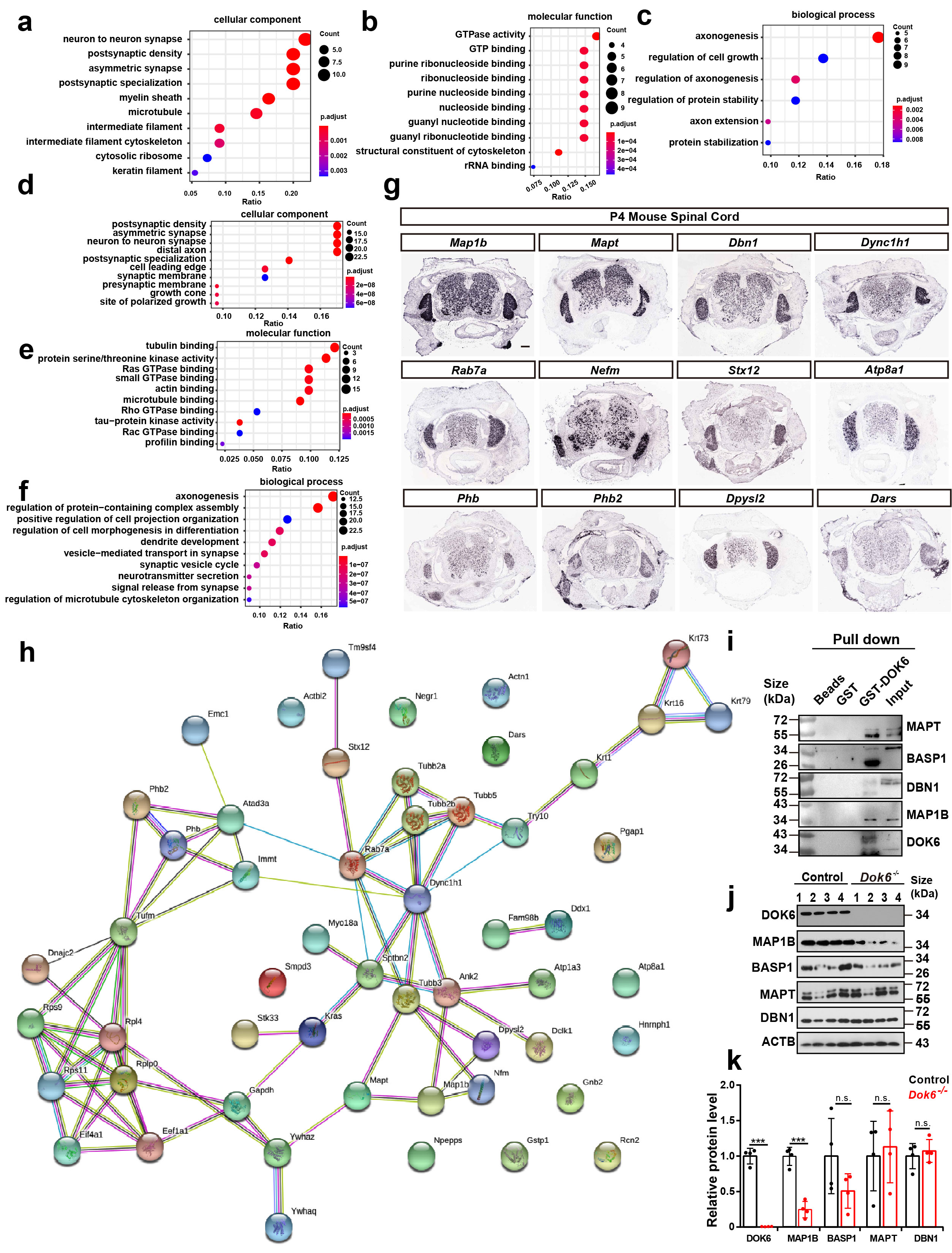


**Figure S5. Analyze the regulatory networks involved in DOK6 through the protein-protein interaction.**

**(a)** Cellular components were analyzed using mass spectrometry data from GST pulldowns. **(b)** Molecular function was analyzed using mass spectrometry data from GST pulldowns. **(c)** Biological processes were analyzed with mass spectrometry data from GST pulldowns. **(d)** Cellular components were analyzed with phosphoproteomic profiling data. **(e)** Molecular function was analyzed with phosphoproteomic profiling data. **(f)** Biological processes were analyzed with phosphoproteomic profiling data. **(g)** RNA *in situ* hybridization data from the were plotted, including *Map1b, Mapt, Dbn1, Dync1h1, Rab7a, Nefm, Stx12, Atp8a1, Phb, Phb2, Dpysl2, and Dars* (lower panel). **(h)** The straight line between the circle nodes represents the interaction between the two proteins, and DOK6 showed an important role in microtube and axonal transport. The *p* value is 1.43e^-12^ (upper panel). **(i)** Verification of four putative proteins (MAP1B, MAPT, BASP1, DBN1) interacting with DOK6 using GST pull-down. **(j-k)** Western blot analysis of MAP1B, BASP1, MAPT, and DBN1 in sciatic nerves from P3 control and *Dok6^-/-^* mice. β-actin served as a loading control. Data are presented as the mean ± SD, ****p < 0. 001.unpaired* t test between different lines of mice.


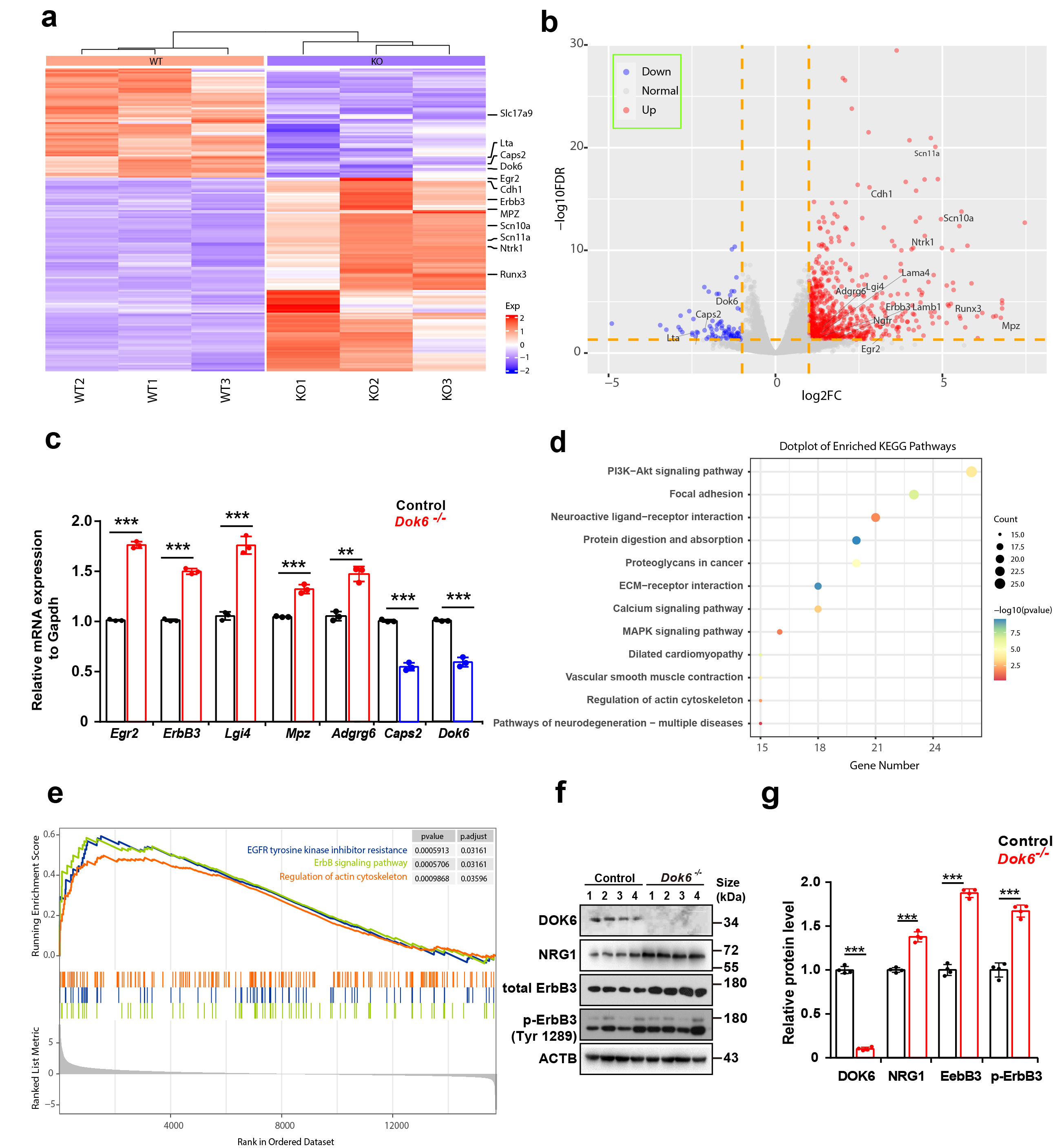


**Figure S6. The expression of myelination-related genes is altered in *Dok6* mutant sciatic nerves at P3. (a)** Heat map of a microarray analysis depicting the top 125 up-regulated and all 84 down-regulated genes in sciatic nerves of 3 *Dok6* mutant mice (Mut) compared to littermate controls (WT) at P3. **(b)** Volcano plot showing up or down-regulated genes detected in control and *Dok6^-/-^* groups. **(c)** qRT-PCR of P3 sciatic nerves from *Dok6* mutant versus WT mice. **(d)** KEGG pathway analysis of differentially expressed genes between *Dok6^-/-^* and control groups. **(e)** Ridgeline plot of GSEA results for KEGG pathway analysis. **(f and g)** Western blot analysis of NRG1, ErbB3 Tyr 1289 phosphorylation (pErbB3) and total ErbB3 in sciatic nerves from P3 control and *Dok6^-/-^* mice. β-actin served as a loading control. Data are presented as the mean ± SD, ***p < 0.01*, ****p < 0. 001.unpaired* t test between different lines of mice.


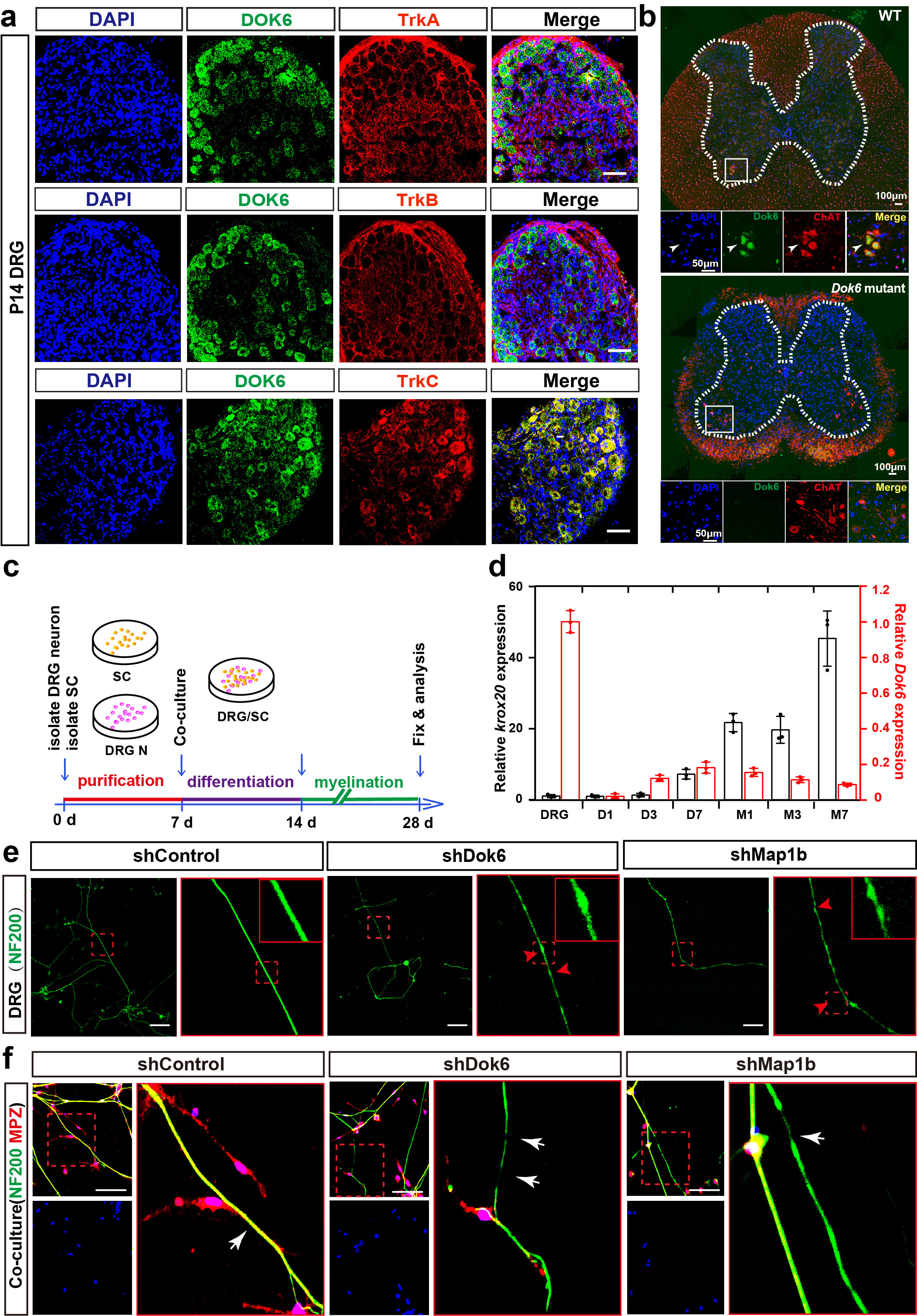


**Figure S7. DOK6 is expressed in specific types of neurons and mediates peripheral neuronal degeneration through the MEK/ERK signaling cascade.** (**a)** Immunofluorescence co-expression of DOK6 with TrkA, TrkB, and TrkC in DRG neurons of P14 mice, Scale bar, 100 μm. **(b)** Co-labeling of Dok6 and ChAT in cholinergic motoneuron-positive cells in the anterior horn of the spinal cord of P14 mice. Blue: DAPI, green: Dok6; Red: ChAT. Arrows indicate neuronal cells colocalizing Dok6 and ChAT. (figure scale bar, 100μm, the amplification figure scale bar, 50μm). **(c)** Schematic timeline diagram of the cell coculture system. **(d)** The expression of *Dok6* and *Krox20* (an SC marker) was tested in the coculture system. D, days in differentiation medium; M, days in myelination medium. **(e)** NF200 immunostaining in sham, *Dok6-*shRNA- and *Map1b-*shRNA lentivirus-infected DRG neurons. The axon was detected after 5 days of cell culture. The red arrow indicates the torpedo-like degenerated axon structure. Scale bar, 100 μm. **(f)** Immunocytochemistry of cocultured DRG neurons and SCs after infection with sham, *Dok6-*shRNA, and *Map1b-*shRNA lentiviruses. The effects of DOK6 and MAP1B on myelination were determined by MPZ staining (red). The arrowhead indicates to unmyelinated axons which are not protected by the myelin sheath. Scale bar, 100 μm.

**Supplementary Table**

**Table S1.** **Correlation between *Dok6*** **genotype and penetrance of the various phenotypes**

| Genotypes | Phenotypes | |
| --- | --- | --- |
| *Dok6* | Aberrant hindlimb | Abnormal posture |
| (+/+) | 0% (0/232) | - |
| (+/-) | 3.9% (12/305) | - |
| (-/-) | 31.1% (71/228) | 2.6% (6/228) |

**Table S2. *Dok6* mutants and conditional *Dok6* knockout mice** **genotyping primers**

| Primer | Sequence |
| --- | --- |
| Dok6 3loxp-F | TGGAGAGCTCTCTTTGTACAAGA |
| Dok6 3loxp-R | TGCAGGTTCAAACGACAAACTGC |
| Dok6 Frt-F | TACCTGGGAGATGTTCAGAATCAGC |
| Dok6 Frt-R | TGCAGGTTCAAACGACAAACTGC |
| Dok6 Aloxp-F | TGGAGAGCTCTCTTTGTACAAGA |
| Dok6 Aloxp-R | GGATAGTATGTGTGCTACATACAGT |
| Cre-F | TCGATGCAACGAGTGATGAG |
| Cre-R | TTCGGCTATACGTAACAGGG |

**Table S3. *In situ* hybridization** **probes primers**

| Primer | Sequence |
| --- | --- |
| Dok6-F | CGAGCTCGCTGCAGACAAGCTTGACTG |
| Dok6-R | CGGTACCTTGAATGAGGCTGGAGCTATAG |
| TrkA-F | GGGGTACCCCATGTCGATGACAGACGTGCTCAG |
| TrkA -R | CCAAGCTTGGGCTTTCAGCCACCAGAGTGG |
| TrkB-F | GCTCTAGAGGACACGCACTCCGACTGA |
| TrkB-R | CGGAATTCATGGAATGCA CCAGTGGTGAT |
| TrkC-F | GCTCTAGAGAGTCTGATGCGAGCCCTAC |
| TrkC-R | CGGAATTCTGTGGTGAACTTCCGGTACA |
| CGRP-F | GGGGTACCCCATGGGCTTCCTGAAGTTCTCCC |
| CGRP -R | CCAAGCTTGGGGCCTGAAGGTCCCTGCGG |
| RET-F | GGGGTACCCCGGCCACAGCCTTCCGTCTG |
| RET-R | GCTCTAGAGCCATGTGGAAGGGAGGGAGCG |
| PV-F | GGGGTACCCCATGTCGATGACAGACGTGCTCAG |
| PV-R | CCAAGCTTGGGCTTTCAGCCACCAGAGTGG |

**Table S4.** **qRT-PCR primers**

| Primer | Sequence |
| --- | --- |
| Dok6-F | CGAGCTCGCTGCAGACAAGCTTGACTG |
| Dok6-R | CGGTACCTTGAATGAGGCTGGAGCTATAG |
| Krox20-F | GCCAAGGCCGTAGACAAAATC |
| Krox20-R | CCACTCCGTTCATCTGGTCA |
| S100β-F | TGGTTGCCCTCATTGATGTCT |
| S100β-R | CCCATCCCCATCTTCGTCC |
| Tubb3-F | TAGACCCCAGCGGCAACTAT |
| Tubb3-R | GTTCCAGGTTCCAAGTCCACC |
| Mbp-F | GGCGGTGACAGACTCCAAG |
| Mbp-R | GAAGCTCGTCGGACTCTGAG |
| Mpz-F | CGGACAGGGAAATCTATGGTGC |
| Mpz-R | TGGTAGCGCCAGGTAAAAGAG |
| ErbB3-F | ATGCGCCCATTTTCGTGATG |
| ErbB3-R | CTGGTCCCTTACACCCTTGG |
| LGI4-F | CACACGCTACATTGGGGACTC |
| LGI4-R | AGCCCCTTGTCAGGCTCAAG |
| EGR2-F | TGGTGTGTGTGGTGGTTGTT |
| EGR2-R | TGTTGATCATGCCATCTCCG |
| CAPS2-F | TAC TGC TTT CCC TTC GGA CG |
| CAPS2-R | TCT GCG GGC CCT TCT ATT TT |
| Gpr126-F | CCAAAGTTGGCAATGAAGT |
| Gpr126-R | GCTGGATCAGGTAGGAACCA |
| Gapdh-F | AGGTCGGTGTGAACGGATTTG |
| Gapdh-R | TGTAGACCATGTAGTTGAGGTCA |

**Table S5.** **GST pull-down followed by mass spectrometry and identified 55 proteins**

| Protein Name | MW (Da) | Main Function | PMID |
| --- | --- | --- | --- |
| Gm10481 | 15535 | oxidoreductase activity | 21873635 |
| Vime | 53688 | negative regulation of neuron projection development | 11487638 |
| Basp1 | 22087 | [cell differentiation](https://www.ebi.ac.uk/QuickGO/term/GO:0072112) | 19050011 |
| GstP1 | 23609 | [regulation of ERK1 and ERK2 cascade](https://www.ebi.ac.uk/QuickGO/term/GO:0070372) | 10945608 |
| Kras | 21656 | [cell population proliferation](https://www.ebi.ac.uk/QuickGO/term/GO:0008284), MAPK[, cell population proliferation](https://www.ebi.ac.uk/QuickGO/term/GO:0008284) | 24058167 |
| Tubb5 | 49671 | [structural constituent of cytoskeleton](https://www.ebi.ac.uk/QuickGO/term/GO:0005200), [microtubule cytoskeleton organization](https://www.ebi.ac.uk/QuickGO/term/GO:0000226) | 15121885 |
| Tubb2a | 49907 | [microtubule cytoskeleton organization](https://www.ebi.ac.uk/QuickGO/term/GO:0000226), [neuron migration](https://www.ebi.ac.uk/QuickGO/term/GO:0001764) | 21873635 |
| Tubb2b | 49953 | [structural constituent of cytoskeleton](https://www.ebi.ac.uk/QuickGO/term/GO:0005200) | 21873635 |
| Tubb3 | 50419 | proper axon guidance | 20074521 |
| Hist | 11367 | [DNA binding](https://www.ebi.ac.uk/QuickGO/term/GO:0003677) | 17193262 |
| Phb2 | 33296 | [positive regulation of ERK1 and ERK2 cascade](https://www.ebi.ac.uk/QuickGO/term/GO:0070374), [cell migration](https://www.ebi.ac.uk/QuickGO/term/GO:0016477) | 32320388 |
| Phb | 29820 | [DNA biosynthetic process](https://www.ebi.ac.uk/QuickGO/term/GO:0071897) | 20074521 |
| Ywhaz | 27771 | [protein domain specific binding](https://www.ebi.ac.uk/QuickGO/term/GO:0019904) | 12176995 |
| Rplp0 | 34216 | [ribosomal large subunit assembly](https://www.ebi.ac.uk/QuickGO/term/GO:0000027) | 21873635 |
| Actbl2 | 42004 | [axonogenesis](https://www.ebi.ac.uk/QuickGO/term/GO:0007409) | 21873635 |
| Rab7a | 23490 | [intracellular protein transport](https://www.ebi.ac.uk/QuickGO/term/GO:0006886) | 11042173 |
| Rps9 | 22591 | [positive regulation of translational fidelity](https://www.ebi.ac.uk/QuickGO/term/GO:0045903) | 21873635 |
| Eef1a | 50114 | [translation](https://www.ebi.ac.uk/QuickGO/term/GO:0006412) | 21873635 |
| Stx12 | 31195 | [intracellular protein transport](https://www.ebi.ac.uk/QuickGO/term/GO:0006886) | 21873635 |
| Hnrnph1 | 49199 | [cellular response to interleukin-7](https://www.ebi.ac.uk/QuickGO/term/GO:0098761) | 29581031 |
| Rps11 | 18431 | [structural constituent of ribosome](https://www.ebi.ac.uk/QuickGO/term/GO:0003735) | 21873635 |
| Ywhaq | 27778 | [protein domain specific binding](https://www.ebi.ac.uk/QuickGO/term/GO:0019904) | [12446771](http://dx.doi.org/10.1128/mcb.22.24.8514-8526.2002) |
| Gnb2 | 37331 | [G protein-coupled receptor signaling pathway](https://www.ebi.ac.uk/QuickGO/term/GO:0007186) | 21873635 |
| Krt16 | 51606 | [intermediate filament cytoskeleton organization](https://www.ebi.ac.uk/QuickGO/term/GO:0045104) | 12445204 |
| Krt73 | 58911 | specific component of keratin intermediate filaments | 24667173 |
| Tufm | 49508 | [mitochondrial translational elongation](https://www.ebi.ac.uk/QuickGO/term/GO:0070125) | 21873635 |
| Krt1 | 65606 | [negative regulation of inflammatory response](https://www.ebi.ac.uk/QuickGO/term/GO:0050728) | 23132931 |
| Krt79 | 57552 | cytoskeletal and microfibrillar keratin | 27573346 |
| Rcn2 | 37271 | binds calcium | 21873635 |
| Nefm | 95916 | [regulation of axon diameter](https://www.ebi.ac.uk/QuickGO/term/GO:0031133), [axo-dendritic transport](https://www.ebi.ac.uk/QuickGO/term/GO:0008088) | 10461886 |
| Dpysl2 | 62278 | [axon guidance](https://www.ebi.ac.uk/QuickGO/term/GO:0007411), [regulation of neuron projection development](https://www.ebi.ac.uk/QuickGO/term/GO:0010975) | 24227739 |
| Myo18a | 232755 | [positive regulation of opsonization](https://www.ebi.ac.uk/QuickGO/term/GO:1903028) | 21123169 |
| Immt | 83678 | [mitochondrial calcium ion homeostasis](https://www.ebi.ac.uk/QuickGO/term/GO:0051560) | 20880836 |
| Atp8a1 | 131413 | [cell migration](https://www.ebi.ac.uk/QuickGO/term/GO:0030335), [transmembrane transport](https://www.ebi.ac.uk/QuickGO/term/GO:0055085) | [20224745](http://dx.doi.org/10.2147/jrlcr.s3773) |
| Atad3a | 66742 | [mitochondrion organization](https://www.ebi.ac.uk/QuickGO/term/GO:0007005) | 21873635 |
| Smpd3 | 71197 | [regulation of protein phosphorylation](https://www.ebi.ac.uk/QuickGO/term/GO:0001932) | 22383528  29725009 |
| Fam98b | 45349 | [protein methylation](https://www.ebi.ac.uk/QuickGO/term/GO:0006479) | 28040436 |
| Mapt | 76243 | [neuron projection development](https://www.ebi.ac.uk/QuickGO/term/GO:0031175) | 21873635 |
| Negr1 | 37900 | [neuron projection development](https://www.ebi.ac.uk/QuickGO/term/GO:0010976) | 22844493 |
| Rpl4 | 47154 | [RNA binding](https://www.ebi.ac.uk/QuickGO/term/GO:0003723) | 21873635 |
| Map1b | 270255 | [cytoskeletal regulatory protein binding](https://www.ebi.ac.uk/QuickGO/term/GO:0005519), [axonogenesis](https://www.ebi.ac.uk/QuickGO/term/GO:0007409), [axon regeneration](https://www.ebi.ac.uk/QuickGO/term/GO:0014012) | 11581286 |
| Actn1 | 103068 | [actin filament bundle assembly](https://www.ebi.ac.uk/QuickGO/term/GO:0051017) | 16043482 |
| Emc1 | 111759 | -- | -- |
| Pgap1 | 104578 | [anterior/posterior axis specification](https://www.ebi.ac.uk/QuickGO/term/GO:0009948) | 10529425 |
| Ank2 | 426261 | [regulation of cation channel activity](https://www.ebi.ac.uk/QuickGO/term/GO:2001257) | 20610380 |
| Ddx1 | 82500 | [positive regulation of myeloid dendritic cell cytokine production](https://www.ebi.ac.uk/QuickGO/term/GO:0002735) | 21703541 |
| Dync1h1 | 532045 | [cytoplasmic microtubule organization](https://www.ebi.ac.uk/QuickGO/term/GO:0031122) | [21873635](http://dx.doi.org/10.1093/bib/bbr042) |
| Npepps | 103325 | [proteolysis](https://www.ebi.ac.uk/QuickGO/term/GO:0006508) | [21873635](http://dx.doi.org/10.1093/bib/bbr042) |
| Eif4a1 | 46154 | [cytoplasmic translational initiation](https://www.ebi.ac.uk/QuickGO/term/GO:0002183) | 21873635 |
| Sptbn2 | 270923 | [postsynapse organization](https://www.ebi.ac.uk/QuickGO/term/GO:0099173) | 22090485 |
| Atp1a3 | 111692 | [cellular sodium ion homeostasis](https://www.ebi.ac.uk/QuickGO/term/GO:0006883) | 19376779 |
| Dclk1 | 84153 | [axonogenesis](https://www.ebi.ac.uk/QuickGO/term/GO:0007409), [neuron migration](https://www.ebi.ac.uk/QuickGO/term/GO:0001764) | 16387638 |
| Try10 | 26221 | [proteolysis](https://www.ebi.ac.uk/QuickGO/term/GO:0006508) | 21873635 |
| Tm9sf4 | 74693 | [protein localization to membrane](https://www.ebi.ac.uk/QuickGO/term/GO:0072657) | 21873635 |
| Dars | 57147 | [RNA binding](https://www.ebi.ac.uk/QuickGO/term/GO:0003723) | 21873635 |
